# Genetically Based Location from Triploid Populations and Gene Ontology of a 3.3-Mb Genome Region Linked to Alternaria Brown Spot Resistance in Citrus Reveal Clusters of Resistance Genes

**DOI:** 10.1371/journal.pone.0076755

**Published:** 2013-10-08

**Authors:** José Cuenca, Pablo Aleza, Antonio Vicent, Dominique Brunel, Patrick Ollitrault, Luis Navarro

**Affiliations:** 1 Centro de Protección Vegetal y Biotecnología, Instituto Valenciano de Investigaciones Agrarias. Moncada, Valencia, Spain; 2 Etude du Polymorphisme des Genomes Vegetaux, Institut National de la Recherche Agronomique, Évry, France; 3 BIOS Department, Amélioration Génétique des Espèces à Multiplication Végétative. Centre de Coopeération Internationale en Recherche Agronomique pour le Développement, Montpellier, France; USDA-ARS-SRRC, United States of America

## Abstract

Genetic analysis of phenotypical traits and marker-trait association in polyploid species is generally considered as a challenge. In the present work, different approaches were combined taking advantage of the particular genetic structures of 2n gametes resulting from second division restitution (SDR) to map a genome region linked to Alternaria brown spot (ABS) resistance in triploid citrus progeny. ABS in citrus is a serious disease caused by the tangerine pathotype of the fungus *Alternaria alternata*. This pathogen produces ACT-toxin, which induces necrotic lesions on fruit and young leaves, defoliation and fruit drop in susceptible genotypes. It is a strong concern for triploid breeding programs aiming to produce seedless mandarin cultivars. The monolocus dominant inheritance of susceptibility, proposed on the basis of diploid population studies, was corroborated in triploid progeny. Bulk segregant analysis coupled with genome scan using a large set of genetically mapped SNP markers and targeted genetic mapping by half tetrad analysis, using SSR and SNP markers, allowed locating a 3.3 Mb genomic region linked to ABS resistance near the centromere of chromosome III. Clusters of resistance genes were identified by gene ontology analysis of this genomic region. Some of these genes are good candidates to control the dominant susceptibility to the ACT-toxin. SSR and SNP markers were developed for efficient early marker-assisted selection of ABS resistant hybrids.

## Introduction

Alternaria brown spot (ABS) is a serious disease that induces necrotic lesions on fruit and young leaves, defoliation and fruit drop in susceptible citrus genotypes [1]. The disease was first observed in Australia in 1903 on the 'Emperor' mandarin [2], and was subsequently detected in citrus-growing regions in America, the Mediterranean Basin, South Africa, Iran and China [3-5]. In Spain, the disease was first detected in 1998 [6], and it is currently widespread in all citrus-growing areas, affecting mainly 'Fortune' and 'Nova' mandarin hybrids. The disease is caused by the tangerine pathotype of the fungus *Alternaria alternata* (Fr.) Keissl, which carries a gene cluster (*ACTT*) located in a small (<2.0 Mb) conditionally dispensable chromosome responsible for ACT-toxin biosynthesis [7]. This host-specific toxin is released during the germination of conidia, rapidly affecting the plasma membrane integrity of susceptible host cells [8]. There is also indirect evidence suggesting the presence of toxin receptors in susceptible citrus genotypes [9]. In addition, recent studies indicate that the mitigation of reactive oxygen species (ROS) produced by the host plants is essential for pathogenicity [10]. The pathogen sporulates on affected tissues, and conidia are disseminated by air currents and rain splash. Warm temperatures and prolonged wetness on the tree are required for infection. The incubation period is very short, and lesions are visible just 1 or 2 days after infection is initiated, due to the rapid effects of the ACT-toxin [11]. The disease causes severe epidemics in humid areas, as well as in semi-arid regions, due to its environmental flexibility [4]. Currently, ABS control is primarily based on the application of fungicides. Sprays must be scheduled to protect susceptible organs during the critical periods for infection. Depending on the climate of the region and the susceptibility of the cultivar, between four and ten fungicide sprays per year are needed to produce quality fruit for the fresh market [12-14].

Despite this large number of sprays, disease control is not always satisfactory, and cultivation of susceptible cultivars such as the 'Fortune' mandarin has declined significantly in Spain during recent years. In addition, systematic application of fungicides for ABS control over many years may create environmental problems and public health concerns [15]. Moreover, in the context of the changing global climate, plant breeding is especially focused on improving resistance to biotic and abiotic stresses.

Several studies have been carried out to determine the resistance or susceptibility to ABS in citrus genotypes [16-21]. Although there are some discrepancies among the results of these studies, resistance is clearly present in ‘clementine’ (*Citrus clementina* Hort. ex Tan), ‘Willowleaf’ (*C. deliciosa* Ten) and ‘satsuma’ (*C. unshiu* Mark) mandarins. Susceptibility has also been well established for ‘Dancy’ (*C. tangerina* Hort. ex Tan) and ‘Fortune’ (supposed *C. clementina* Hort. ex Tan × *C. tangerina* Hort. ex Tan) mandarins; ‘Orlando’, ‘Minneola’ and ‘Nova’ tangelos (mandarin × grapefruit hybrids); and the ‘Murcott’ tangor (supposed mandarin × sweet-orange hybrid). Other cultivars such as the ‘Ellendale’ tangor and some sweet oranges and grapefruits have been characterised as sensitive or resistant by different authors. From diploid progeny analysis, it has been proposed that inheritance of ABS resistance in citrus is controlled by a single recessive allele [19,22]. Resistance to the strawberry and pear *Alternaria* pathotypes, which produce toxins structurally analogous to those of the tangerine pathotype, as well as resistance to the apple pathotype, is controlled in the same way, by a single recessive allele [9]. Therefore, resistant cultivars are considered to be recessive homozygous for this locus, whereas susceptible cultivars could be heterozygous or homozygous dominant.

Diploidy is the general rule in *Citrus* and related genera; however, polyploidy manipulation is currently widely used in triploid citrus breeding programs aimed at developing new seedless mandarin cultivars [23]. Many of these breeding programs [24-33] use ABS-susceptible cultivars as parents, due to their utility with regard to other important traits (fruit quality, maturing period, production) and particular reproductive biology (monoembryony, high rate of triploid production). The inheritance and efficient selection of resistance to ABS is therefore of central importance to triploid mandarin breeding projects.

Genetic analysis of phenotypical traits and marker-trait association in polyploid species is generally considered as a challenge due to complex segregation, dosage effects and potential non Mendelian inheritance associated with epigenetic variations.

The main factor affecting trait inheritance in triploid families is the strategy used for triploid breeding [23], with significant differences between the sexual polyploidisation approach (2*x* × 2*x* crosses with unreduced -2*n*- gamete formation) and interploid crosses (2*x* × 4*x* or 4*x* × 2*x*). Indeed, the choice of strategy affects the transmission of parental heterozygosity to the diploid gamete.

In sexual polyploidisation, two factors affect the transmission of parental heterozygosity to the offspring: the mechanism of 2*n* gamete formation (i.e., first-division restitution [FDR] or second division restitution [SDR]) and the genetic distance from the locus of interest to the centromere [34]. Therefore, Half-Tetrad Analysis (HTA) based on 2*n* gametes is an efficient means of genetic mapping [35-37]. In 2*x* × 2*x* citrus crosses, the diploid (unreduced) gamete is transmitted by the female parent [38,39]. SDR has been proposed for diploid megagametophyte development in ‘Clementine’ [40] and ‘Fortune’ mandarins [41], whereas FDR has been reported in sweet oranges [42]. Recent studies have revealed that SDR is the main mechanism involved in unreduced gamete formation in the majority of citrus cultivars [43]. For interploid crosses, most of the tetraploid parents used in citrus breeding arise from chromosome doubling in nucellar cells of apomictic diploid parents [44]. Because mandarins are one of the ancestral species of cultivated citrus [45], doubled-diploid mandarins should be considered as autotetraploid, and tetrasomic inheritance should be expected [46]. In such a situation, the frequency of diploid gametes that receive a locus in heterozygosis from the tetraploid parent varies between 0.55 and 0.66, depending on the double-reduction frequency [47].

In addition to the particular transmission of chromosome fragments and parental heterozygosity, the phenotypic trait inheritance in polyploids can be affected by dosage effects [48,49] and even by neoregulation of gene expression due to epigenomic reformatting [50,51], eventually leading to non- Mendelian segregation. Moreover, polyploidy induces morphological variations in leaves and fruits [52-54] that should affect fungus colonisation. In this context, no data have yet been published regarding the inheritance of ABS resistance in triploid progenies.

Due to its direct applicability in marker-assisted selection, the identification of molecular markers linked to phenotypic variation, e.g., related to disease resistance, is a key step in most breeding programs. Bulked segregant analysis [BSA [55]] can be used to identify molecular markers in a genomic region associated with a specific phenotype rapidly. This method is based on linkage disequilibrium between the gene and linked markers in segregating progeny, and the genetic linkage between markers and the causal gene is determined by differences in marker-allele frequencies between resistant and susceptible bulks. For characters controlled by one or a few genes, BSA is an effective technique for detecting alleles linked to phenotypes in a large sample of progeny at a relatively low cost, where the only requirement is that the genotyping technique and molecular markers utilised provide quantitative measurements of allelic frequencies [56]. This approach should be optimised by coupling BSA with a high-throughput genotyping method using markers covering the whole genome. Genome-wide association studies of pooled DNA samples have been valuable tools in the fast, scalable and economical identification of candidate single nucleotide polymorphisms (SNPs) associated with a phenotype [57-64]. In citrus, very large SNP resources are becoming available from extensive citrus sequencing projects [65-68]; meanwhile, new technologies have been developed for very rapidly genotyping large numbers of SNPs in DNA samples. One such technology is the GoldenGate assay from Illumina™ [59,69,70], which proved useful in citrus by allowing mapping of 677 SNP markers onto the ‘Clementine’ consensus map [71].

The objectives of this study were (i) to confirm Mendelian monolocus inheritance of ABS resistance in triploid progenies, by analysing the segregation of resistance in different interploid crosses, and to confirm the dominance of susceptibility by analysing segregation of resistance in progeny produced by sexual polyploidisation and interploid hybridisation; (ii) to locate the chromosome region associated with the ABS resistance using a genome scan assay coupled with BSA, followed by targeted genetic mapping by HTA in triploid progenies arising from 2n gametes; and (iii) to identify candidate resistance genes in the located region, taking advantage of the recently released reference whole genome sequence of *C. clementina* [72]. A more applied objective was identification of molecular markers for marker-assisted selection (MAS) in citrus breeding programs.

## Materials and Methods

### Plant material

Seven hundred and fourteen triploid hybrids arising from four 2*x* × 2*x* crosses, three 2*x* × 4*x* crosses and their parents were evaluated for field and *in vitro* infection by *A. alternata*. Parental genotypes included in the citrus germplasm bank and hybrids were grown at the ‘Instituto Valenciano de Investigaciones Agrarias’ (I.V.I.A.) orchards in Moncada, Valencia, Spain. The plantings were very dense, with conditions very favourable for the development of ABS infection.

Information about parental accessions, their origin, ABS phenotype and references are shown in Table 1. The genetic configuration of the *ABSr* locus (‘A’, dominant susceptible allele; ‘a’, recessive resistant allele) for each parental accession (also given in the table) has been deduced from information about ABS resistance/susceptibility of diploid genotypes, their pedigree and segregation data at the diploid level, under the hypothesis of single locus inheritance. The tetraploids ‘Nova’ and ‘Orlando’ resulted from chromosome stock doubling of the Nova and Orlando diploids, respectively [44]. The diploid lines are considered to be ‘Aa’ at the *ABSr* locus; therefore, the genotypes of the two tetraploid parents should be ‘AAaa’.

**Table 1:** Parental genotypes used in this study, phenotypic information on ABS resistance and deduced *ABSr* locus genotyping.

**Genotype**	**Origin**	**Phenotype**	**Reference**	***ABSr* locus genotype**
‘Fortune’	*C. clementina* X *C. tangerina*	S	[6,20]	Aa
‘Minneola’	*C. paradisi*’ X *C. tangerina*	S	[114-117]	AA
‘Orlando’	*C. paradisi* X *C. tangerina*	S	[22,115]	2x: Aa; 4x: AAaa
‘Nova’	*C. clementina* X (*C. paradisi* X *C. tangerina*)	S	[19]	2x: Aa; 4x: AAaa
‘Murcott’	(unknown)	S	[19-115]	Aa
‘Willowleaf’	*C. deliciosa*	R	[115]	aa
‘Clemenules’ ‘Clementina Fina’	*C. clementina*	R	[20,22,74,115,117]	aa
‘Nadorcott’	‘Murcott’ X unknown	R	Our unpublished data	aa

(S) Susceptible phenotype; (R) Resistant phenotype; (A) Susceptible allele; (a) Resistant allele

Three of the 2*x* × 2*x* crosses share ‘Fortune’ as the female parent, with ‘Willowleaf’ mandarin (93 hybrids), ‘Minneola’ tangelo (127 hybrids) and ‘Murcott’ (148 hybrids) as male parents. The other 2*x* × 2*x* cross was ‘clementina Fina’ × ‘Nadorcott’ (50 hybrids). Details on procedures for establishing the triploid populations from 2*x* × 2*x* crosses by embryo rescue and triploid selection by flow cytometry can be found in [28].

Two of the 2*x* × 4*x* crosses share ‘Orlando 4x’ as the male parent, with ‘Clemenules’ (180 hybrids) and ‘Fortune’ (116 hybrids) as female parents. The other 2*x* × 4*x* population was ‘Clemenules’ × ‘Nova 4x’ (100 hybrids). Information about procedures for establishing the 2*x* × 4*x* populations can be found in [30].

Moreover, five additional triploid populations arising from 2*x* × 2*x* and 2*x* × 4*x* crosses (114 hybrids) were also evaluated for ABS resistance to extend the experiments to other genetic backgrounds. Due to the relatively low number of triploid hybrids within each population, the resultant data have been included as supplementary material (Table S1).

### Evaluation of ABS resistance

Previous studies in diploid genotypes have shown a range of susceptibility level among citrus germplasm, but suggest that immune response could be controlled by a single recessive allele [19,22]. In the present study, genotypes have been considered as resistant if no symptoms have been observed neither under field evaluations nor leaf inoculations. Therefore in this study, as in the previous ones at diploid level [19-22], the resistant phenotype corresponds to immune symptom.

#### Field evaluation

Symptoms of *A. alternata* were evaluated for all genotypes on trees grown at the I.V.I.A. orchards in spring, when young leaves are more susceptible to ABS and environmental conditions are highly favourable for infection [15]. Presence or absence of ABS symptoms on the leaves was recorded in a qualitative manner. For each tree, observations were carried out over three consecutive years (2010, 2011 and 2012).

#### *In vitro* inoculation of detached leaves

**Inoculum production:** A virulent single-spore isolate of *A. alternata* (IVIA-A005) isolated from an infected ‘Fortune’ fruit from Valencia (Spain) was used for inoculations. Abundant conidia were obtained by a method adapted from [73]. The isolate was grown on potato dextrose agar (PDA) plates at 25°C in darkness for 8–10 days, illuminated with ﬂuorescent lamps (Philips TLD 18W/33) at 25°C for 8 h to initiate conidiophore formation, and then placed in the dark at 18°C for 12 h. Conidial suspensions were prepared by pouring sterile water over the colonies and gently rubbing the surface with a sterile glass rod. The suspension was ﬁltered through two layers of cheesecloth, and the spore concentration was adjusted to 10^5^ conidia·ml^-1^ with a haemocytometer. Suspensions with conidial germination lower than 90% were discarded.

**Figure 1:**
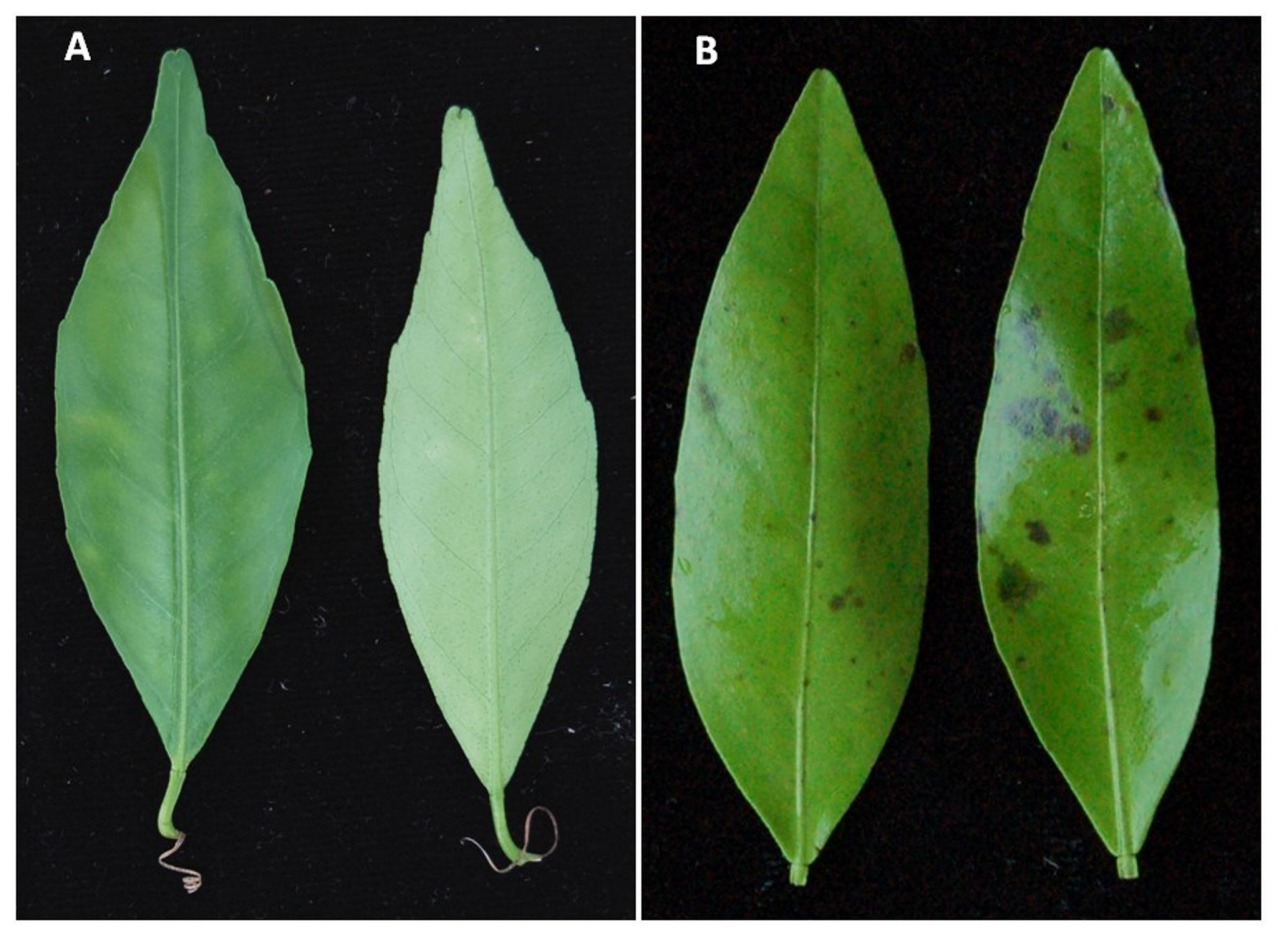
Leaves of resistant genotype ‘Willowleaf’ mandarin (A) and susceptible genotype ‘Fortune’ mandarin (B) showing ABS symptoms 48h after inoculation with a suspension of 10^5^ conidia·ml^-1^.

**Leaf inoculations:** Bioassays were performed immediately after leaf harvest. Young leaves (about 50% developed) were inoculated with 10^5^ conidia·ml^−1^ [74]. This suspension was sprayed over both upper and lower surfaces of each leaflet, using five leaves per genotype. Controls were inoculated by spraying sterile distilled water. Leaves were incubated in a moist chamber in the dark at 27°C, and the results were evaluated 48h after inoculation. In susceptible genotypes, leaf symptoms appear during the second day after inoculations and very clear necrosis induced by the ACT-toxin can be observed after 48h (Figure 1).

A genotype was considered resistant when no symptoms of ABS were observed in any leaf, whereas presence of infection was recorded when a clear symptom of ABS was observed in any leaf. The inoculations were repeated when there was doubt regarding interpretation. The complete experiments were carried out twice during spring of 2010 and twice during spring of 2011.

Two triploid populations derived from 2*x* × 2*x* crosses [‘Fortune’ (‘Aa’) × ‘Minneola’ (‘AA’) and ‘Clementina Fina’ (‘aa’) × ‘Nadorcott’ (‘aa’)] and three triploid populations derived from 2*x* × 4*x* crosses [‘Clemenules’ (‘aa’) × ‘Orlando 4x’ (‘AAaa’), ‘Fortune’ (‘Aa’) × ‘Orlando 4x’ (‘AAaa’) and ‘Clemenules’ (‘aa’) × ‘Nova 4x’ (‘AAaa’)] were phenotyped to compare the expected and observed proportions of resistant and susceptible genotypes, to confirm the monolocus inheritance and dominance of the ABS susceptibility. For the ‘Aa’ × ‘AA’ cross, all segregation progeny are expected to be susceptible to ABS (‘AAA’, ‘AAa or ‘Aaa’), whereas for the ‘aa’ × ‘aa’ cross, all segregation progeny are expected to be ABS resistant (‘aaa’). In case of the ‘aa’ × ‘AAaa’ and ‘Aa’ × ‘AAaa’ crosses, the resistant and susceptible proportions depend on heterozygosity restitution (HR) from the tetraploid parent to the progeny, which varies between 0.55 and 0.66 depending on the double-reduction frequency [47]. Therefore, in these cases, the resistant proportions are expected to be between 0.1667 and 0.225 for the ‘aa’ × ‘AAaa’ cross and between 0.0833 and 0.1125 for the ‘Aa’ × ‘AAaa’ cross (Table 2). χ^2^ tests were conducted on the observed and expected frequencies. In cases of diploid × tetraploid crosses, where expected frequencies are included in an interval (according to the double-reduction frequency), if the observed value was found to be out of the interval, the observed value was compared with the closest value flanking this interval.

**Table 2:** Expected proportions of ABS locus allelic configuration (AAA, AAa, Aaa or aaa) for each population evaluated.

	**SUSCEPTIBLE**	**SUSCEPTIBLE**	**SUSCEPTIBLE**	**TOTAL SUSCEPTIBLE**	**RESISTANT**
**POPULATION**	**AAA**	**AAa**	**Aaa**	**AAA, AAa, Aaa**	**aaa**
**‘Fortune’ X ‘Minneola’ (Aa X AA)**	$\frac{\left(1-HR\right)}{2}$	*HR*	$\frac{\left(1-HR\right)}{2}$	*1*	*0*
**‘Clementina Fina’ X ‘Nadorcott’ (aa X aa)**	*-*	-	-	0	1
**‘Clemenules’ X ‘Orlando 4x’ (aa X AAaa)**	*-*	0.1667-0.225	0.55-0.66	0.775-0.833	0.1667-0.225
**‘Fortune’ X ‘Orlando 4x’ (Aa X AAaa)**	0.0833-0.1125	0.3875-0.4167	0.3875-0.4167	0.8875-0.9167	0.0833-0.1125
**‘Clemenules’ X ‘Nova 4x’ (aa X AAaa)**	*-*	0.1667-0.225	0.55-0.66	0.775-0.833	0.1667-0.225

HR: maternal heterozygosity restitution

### Estimation of the locus-centromere genetic distance under the hypothesis of monolocus inheritance

Two segregating triploid progeny derived from crosses between ‘Fortune’ (‘Aa’) as the female parent and ‘Willowleaf’ (‘aa’) and ‘Murcott’ (‘Aa’) as male parents have been used to estimate the locus-centromere distance. Because SDR is the mechanism leading to unreduced gamete formation in ‘Fortune’ [41], the maternal HR frequency varies between 0 at the centromere to 0.66 if a model of no chromosome interference is assumed. However [41], demonstrated that the Cx(Co)^4^ model assuming partial chromosome interference was better adapted to the observed HR in Fortune 2*n* gametes.

**Figure 2:**
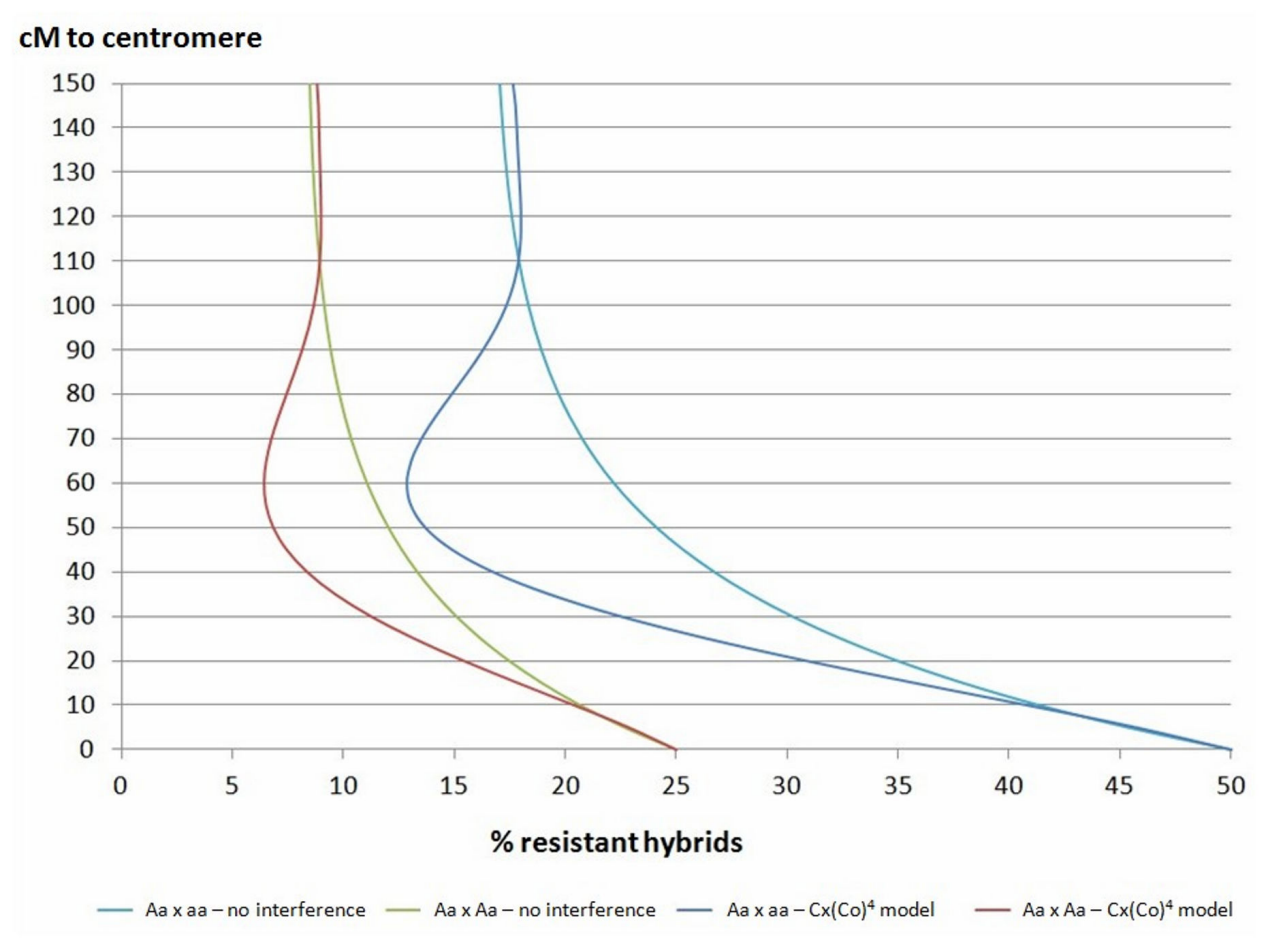
Locus-centromere distance estimated from the proportion of resistant hybrids observed in ‘Aa’ × ‘aa’ and ‘Aa’ × ‘Aa’ crosses under a model of no chromosome interference and the Cx(Co)^4^ model of partial chromosome interference.

The functions for estimating the frequency of diploid gametes that would be heterozygous for a given locus according to its distance from the centromere can be easily modified to estimate the expected genotypic frequency within resultant triploid progeny and even the expected segregation of phenotypic traits with monolocus inheritance. Considering that ABS resistance is a recessive trait controlled by a single locus, susceptible triploid genotypes may have ‘AAA’, ‘AAa’ or ‘Aaa’ allele configurations, whereas resistant triploid genotypes should present only the ‘aaa’ configuration for this locus (Table 3). Therefore, the frequency of resistant genotypes within each population is informative for HR estimation, and therefore for determination of the locus-centromere distance. The relation between centromere distance and percentage of resistant hybrids in controlled progeny have been represented (Figure 2) for the two models of crosses corresponding to the ‘Fortune’ × ‘Willowleaf’ and ‘Fortune’ × ‘Murcott’ crosses (‘Aa’ × ‘aa’ and ‘Aa’ × ‘Aa’, respectively) under two models of chromosome interference (no interference and partial interference). It should be noted that under the Cx(Co)^4^ model of partial chromosome interference, the frequencies of resistant hybrids under 20% and 10% for the ‘Aa’ × ‘aa’ and ‘Aa’ × ‘Aa’ crosses, respectively, can correspond to two different distances from the centromere.

**Table 3:** Expected susceptible and resistant proportions for ‘Fortune’ (‘Aa’) X ‘Willowleaf’ (‘aa’) and ‘Fortune’ (‘Aa’) × ‘Murcott’ (‘Aa’) populations.

	**SUSCEPTIBLE**	**SUSCEPTIBLE**	**SUSCEPTIBLE**	**TOTAL SUSCEPTIBLE**	**RESISTANT**
**POPULATION**	**AAA**	**AAa**	**Aaa**	**AAA, AAa, Aaa**	**aaa**
**‘Fortune’ X ‘Willowleaf’ (Aa x aa)**	*-*	$\frac{\left(1-HR\right)}{2}$	*HR*	$\frac{\left(1+HR\right)}{2}$	$\frac{\left(1-HR\right)}{2}$
**‘Fortune’ X ‘Murcott’ (Aa X Aa)**	$\frac{\left(1-HR\right)}{4}$	$\frac{\left(1-HR\right)}{4}+\frac{HR}{2}=\frac{\left(1+HR\right)}{4}$	$\frac{\left(1-HR\right)}{4}+\frac{HR}{2}=\frac{\left(1+HR\right)}{4}$	$\frac{\left(3+HR\right)}{4}$	$\frac{\left(1-HR\right)}{4}$

HR: maternal heterozygosity restitution

### Bulk segregant analysis coupled with genome scan

BSA [55] has been used to identify genomic regions linked to ABS resistance. To simplify the analysis with only one parental segregation of ABS resistance, the ‘Aa’ × ‘aa’ population (rather than ‘Aa’ × ‘Aa’) was selected. Triploid hybrids from the ‘Fortune’ (‘Aa’) × ‘Willowleaf’ (‘aa’) population yielding conclusive phenotypes (resistant or susceptible) in both field and *in vitro* evaluations were selected for this purpose. Genomic DNA of triploid hybrids and their parents was isolated using the Plant DNeasy kit from Qiagen, Inc. (Valencia, CA, USA), following the manufacturer’s protocol. DNA concentrations were estimated with PicoGreen® and adjusted to 30 ng/µl. Four resistant and four susceptible DNA bulks were established by mixing DNA from five resistant or susceptible hybrids. Each bulk and the parents were genotyped using an Illumina GoldenGate™ array platform, which contains 1536 SNP markers [68]. Six hundred and seventy-seven of these SNPs are mapped in the 'Clementine' reference genetic map [71].

For the mapped markers that were heterozygous in the ‘Fortune’ genotype, we estimated the relative allele signal in each bulk by allelic composition measurement, called the “B allele frequency” (BAF) by Illumina™ [70], using the Illumina® GenomeStudio 2009. The BAF parameter varies between 0 and 1 and is related to the proportion of the B allele versus A+B (SNP genotyping in GoldenGate™ array is diallelic). For pooled samples, this parameter provides useful information on the BAF in the bulk.

ANOVA were performed using BAF information, and the significance of the differentiation between the resistant and susceptible bulks was tested by the F statistic. The pattern of this F parameter along the genome allowed identification of genomic regions with high probability of association with phenotype variation.

### Individual genotyping and mapping of the ABS resistance gene

Ninety-three triploid hybrids for the ‘Fortune’ × ‘Willowleaf’ population and their diploid parents were genotyped using available SSR and SNP markers already mapped [71] in the interval identified by the BSA analysis or developed from the 'Clementine' genomic sequence as described below.

#### New SSR and SNP marker development

We have taken advantage of the recent release of the reference citrus genome sequence (haploid Clementine genome publicly available at www.phytozome.net/clementine) by the International Citrus Genomics Consortium (ICGC) to develop new markers in the genomic region surrounding the SNPs identified by BSA genome scan as linked to ABS resistance. Microsatellites motifs were searched using Sputnik software (http://espressosoftware.com/sputnik/) and new SSR markers were developed and tested for useful polymorphisms. Moreover, 4.47 kb corresponding to four DNA fragments within this region were sequenced in ‘Fortune’ and ‘Willowleaf’ to find SNPs that could be heterozygous in ‘Fortune’ and homozygous in ‘Willowleaf’ mandarin (information on location of the corresponding sequences on the haploid Clementine reference genome and primers used to amplify these DNA fragments is given in Table S2).

#### SSR analyses

Polymerase chain reactions (PCRs) were performed with wellRED oligonucleotides (Sigma-Aldrich®, St Louis, MO, USA) using the following protocol: Mastercycler ep Gradient S (Eppendorf Scientiﬁc Inc., Westbury, NY, USA); reaction volume, 15 µl; 0.8 U Taq polymerase (Fermentas®, Burlington, VT, USA); reaction buffer: 750 mM Tris-HCl (pH 9), 50 mM KCl, 200 mM (NH_4_)_2_SO_4_, 0.001% bovine serum albumin, 0.1 mM of each dNTP, 5 mM MgCl_2_, 3 mM of each primer, 30 ng DNA. The PCR program was as follows: 94°C for 5 min; 40 cycles of 30 s at 94°C, 1 min at 55°C and 30 s at 72°C; ﬁnal elongation 10 min at 72°C. Separation was carried out by capillary gel electrophoresis (CEQ 8000 Genetic Analysis System; Beckman Coulter Inc., Fullerton, CA, USA). Data collection and analysis were carried out using the GenomeLab GeXP (Beckman Coulter Inc.) version 10.0 software.

#### SNP analyses

SNP genotyping was performed by Kbioscience® services, using the KASPar technique. Detailed explanation of specific conditions and reactives can be found in [75].

Assignment of allelic configuration in heterozygous triploid hybrids was carried out using the MAC-PR method for SSR markers [76], or using relative allele signal as proposed by [77] for SNPs genotyped by the KASPar (KBioscience®, UK) technique. Maternal HRs within the triploid progeny were used for *de novo* mapping of the markers in relation to the centromere position, using the Cx(Co)^4^ model for SDR with partial interference [41,78].

Allelic phase of linked marker loci was inferred from the preferential association at the population level between the phenotype (resistant/susceptible) and the maternal alleles. Marker alleles linked with susceptibility were codified as ‘a’ alleles, and those linked with resistance as ‘b’ alleles. The global coherence of this phase attribution was checked by performing a correlation (Pearson’s coefficient) from an individual/loci matrix with values of 1, 0.5, and 0 for the ‘aa’, ‘ab’, and ‘bb’ genotypes, respectively. These correlation values were also used to determine the locations of the various markers in the relative chromosome arms (i.e., on either side of the centromere) in the *de novo* mapping process.

The relative position of the *ABSr* locus and markers were analysed by performing a multiple correspondence analysis (MCA), considering markers as individuals and the various 2*n* gametes as variables. From the previous matrix, we established the qualitative matrix for the factorial analysis by grouping 1 and 0.5 as the same modality (presence of the ‘a’ allele linked with the dominant susceptibility allele in ‘Fortune’) and considering the absence of the ‘a’ allele as the other modality. XLSAT was used to calculate the Pearson’s correlation coefficient and to perform the MCA.

### Gene ontology

All genes encountered within the genomic region between the two markers flanking the estimated location of the *ABSr* locus were searched in the ‘Clementine’ whole genome assembly delivered by the ICGC and publicly available at www.phytozome.net/clementine. The corresponding annotation data were then processed with Blast2GO [79] to provide a global description of the cellular components and biological processes of the genes identified in this genome region.

## Results

### Segregation of ABS resistance in various triploid progeny arising from sexual polyploidisation and interploid crosses

#### Field and *in vitro* evaluation of ABS resistance

Symptoms of ABS were evaluated for all parental accessions and hybrids both from visual inspection of the trees grown at orchards and by *in vitro* inoculations with a conidial suspension of the pathogen. Results obtained for parental genotypes were according to those cited in the literature: ‘Fortune’, ‘Minneola’, ‘Murcott’ and ‘Orlando’ exhibited symptoms of ABS both in the field and *in vitro* in all evaluations; ‘Clemenules’, ‘Clementina Fina’, ‘Willowleaf’ and ‘Nadorcott’ did not exhibit any ABS symptoms on their leaves at any time. Triploid hybrids derived from various evaluated crosses were susceptible (exhibiting typical ABS symptoms) or resistant at proportions depending on the progeny evaluated. No resistant genotypes were found within the ‘Fortune’ × ‘Minneola’ population, whereas all triploid hybrids from the cross between two resistant genotypes (‘Clementina Fina’ × ‘Nadorcott’) were resistant to ABS. Total concordance between field and *in vitro* evaluations was observed for all evaluated populations with the exception of ‘Clemenules’ × ‘Orlando 4x’, where it was over 97% (Table 4).

**Table 4:** Results of field and *in* *vitro* phenotyping for Alternaria brown spot, showing the number of resistant hybrids within each population and the concordance between both types of evaluation.

	**‘Fortune’ X ‘Willoleaf’**	**‘Fortune’ X ‘Murcott’**	**‘Fortune’ X ‘Minneola’**	**‘Clemenules’ X ‘Orlando 4x’**	**‘Clemenules’ X ‘Nova 4x’**	**‘Fortune’ X ‘Orlando 4x’**	**‘Clementina Fina’ X ‘Nadorcott’**
**Number of hybrids evaluated**	93	148	127	180	100	116	50
**Resistant hybrids by field evaluation**	37 (39.78%)	26 (17.57%)	0 (0%)	46 (25.55%)	16 (16%)	12 (10.34%)	50 (100%)
**Resistant hybrids by in vitro evaluation**	37 (39.78%)	26 (17.57%)	0 (0%)	41 (22.78%)	16 (16%)	12 (10.34%)	50 (100%)
**Field-in vitro concordance (%)**	93/93 (100%)	148/148 (100%)	127/127 (100%)	175/180 (97.22%)	100/100 (100%)	116/116 (100%)	50/50 (100%)
**Consensus hybrids evaluated**	93	148	127	175	100	116	50
**Consensus resistant hybrids**	37/93 (39.78%)	26/148 (17.57%)	0/127 (0%)	41/175 (23.43%)	16/100 (16%)	12/116 (10.34%)	50/50 (100%)

#### Inheritance of ABS resistance

Observed resistant proportions within the ‘Fortune’ (‘Aa’) × ‘Minneola’ (‘AA’), ‘Clemenules’ (‘aa’) × ‘Orlando 4x’ (‘AAaa’), ‘Clemenules’ (‘aa’) × ‘Nova 4x’ (‘AAaa’), ‘Fortune’ (‘Aa’) × ‘Orlando 4x’ (‘AAaa’) and ‘Clementina Fina’ (‘aa’) × ‘Nadorcott’ (‘aa’) populations are shown in Table 5. As expected, no resistant genotypes were observed within the ‘Fortune’ × ‘Minneola’ triploid population, whereas no susceptible ones were observed within the ‘Clementina Fina’ × ‘Nadorcott’ population. Regarding the interploid crosses, 41/175 (23.43%) and 16/100 (16%) triploid hybrids were phenotyped as resistant within the ‘Clemenules’ × ‘Orlando 4x’ and ‘Clemenules’ × ‘Nova 4x’ populations, respectively. These values are not significantly different (χ^2^=0.087, p-value=0.769 and χ^2^=0.032, p-value=0.857, respectively) to the closest value of the theoretical interval (16.67–22.5%) under the hypothesis of single locus recessive inheritance of resistance in an ‘aa’ × ‘AAaa’ cross. In the same way, the observed proportion of resistant hybrids in ‘Fortune’ × ‘Orlando 4x’ is within the theoretical interval under the same hypothesis for an ‘Aa’ × ‘AAaa’ cross.

**Table 5:** Expected and observed frequencies of Alternaria brown spot resistant hybrids under the hypothesis of single dominant inheritance within each population and significances of χ^2^ conformity tests.

	**‘Fortune’ X ‘Minneola’ (‘Aa’ X ‘AA’)**	**‘Clemenules’ X ‘Orlando 4x’ (‘aa’ X ‘AAaa’)**	**‘Clemenules’ X ‘Nova 4x’ (‘aa’ X ‘AAaa’)**	**‘Fortune’ X ‘Orlando 4x’ (‘Aa’ X ‘AAaa’)**	**‘Clementina Fina’ X ‘Nadorcott’ (‘aa’ X ‘aa’)**
Number of evaluated hybrids	127	175	100	116	50
Expected resistant proportion (%)	0%	16.67%-22.5%	16.67%-22.5%	8.33%-11.25%	100%
Observed resistant proportion (%)	0%	23.43%	16.00%	10.34%	100%
**χ^2^ test; p-value**	**NS**	**0.087; 0.769 (NS**)	**0.032; 0.857 (NS**)	**WTI**	**NS**

These results confirmed the single dominant inheritance of the ABS susceptibility in triploid populations. Moreover, results of the five additional triploid populations evaluated for ABS resistance (Table S1) also confirm the single recessive inheritance of ABS resistance.

### Estimation of the genetic distance of the ABS resistance locus (ABSr) to the centromere

For the two triploid populations arising from 2*x* × 2*x* crosses (‘Fortune’ × ‘Willowleaf’ and ‘Fortune’ × ‘Murcott’), the proportions of resistant and susceptible hybrids are related to the *ABSr* locus-centromere distance. To estimate the locus-centromere distance, we used a simple cross model in which only the 2*n* gametes segregate for the *ABSr* locus (‘Fortune’ × ‘Willowleaf’). This allows avoiding any eventual bias associated with distorted segregation from the male parent.

The proportion of resistant hybrids in the ‘Fortune’ × ‘Willowleaf’ population was 39.78%, corresponding to an HR estimation value of 0.2043, assuming that ‘aa’ and ‘AA’ 2*n* gametes were equally represented. Only one value for centromere distance is associated with the observed proportion of resistant hybrids when the functions presented in Material and Methods are applied (Figure 2). Moreover, no interference and partial chromosome interference models gave very similar estimates for the centromere distance, which has been estimated to be 10.5 cM.

With such an *ABSr* locus-centromere distance, the expected proportion of the resistant genotype in ‘Fortune’ × ‘Murcott’ progeny (‘Aa’ × ‘Aa’) should be 19.9%. The observed value (17.57%) is not significantly different (χ^2^=0.501), confirming the proximity of the *ABSr* locus to a centromere.

### Bulk segregant analysis coupled with genome scan

**Figure 3:**
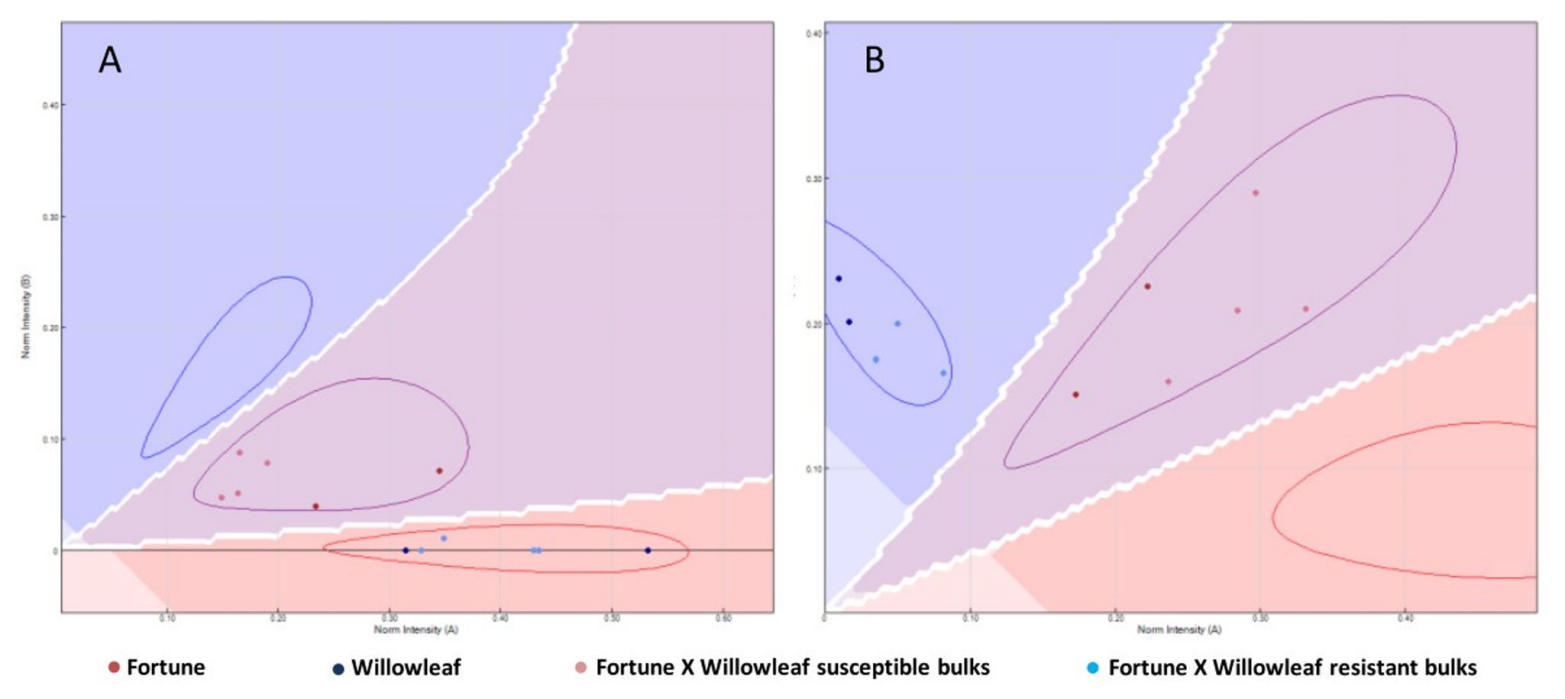
Plot showing Bulked Segregant Analysis results for the CiC3248-06 (A) and CiC6243-03 (B) markers, distinguishing between susceptible and resistant genotypes and bulks.

BSA over the ‘Fortune’ (‘Aa’) × ‘Willowleaf’ (‘aa’) population has been used to identify a genomic region linked to the ABS resistance gene. Four resistant and four susceptible bulks were genotyped for 1536 SNP markers using a GoldenGate™ array platform. Of these, 429 SNP markers were heterozygous for the ‘Fortune’ mandarin and were used to perform ANOVA analyses over relative allele signal for each bulk; significance of the differentiation between the resistant and susceptible bulks was tested by the F statistic. A graphical example for the CiC3248-06 and CiC6243-03 markers, which differentiate resistant and susceptible bulks, is shown in Figure 3.

**Figure 4:**
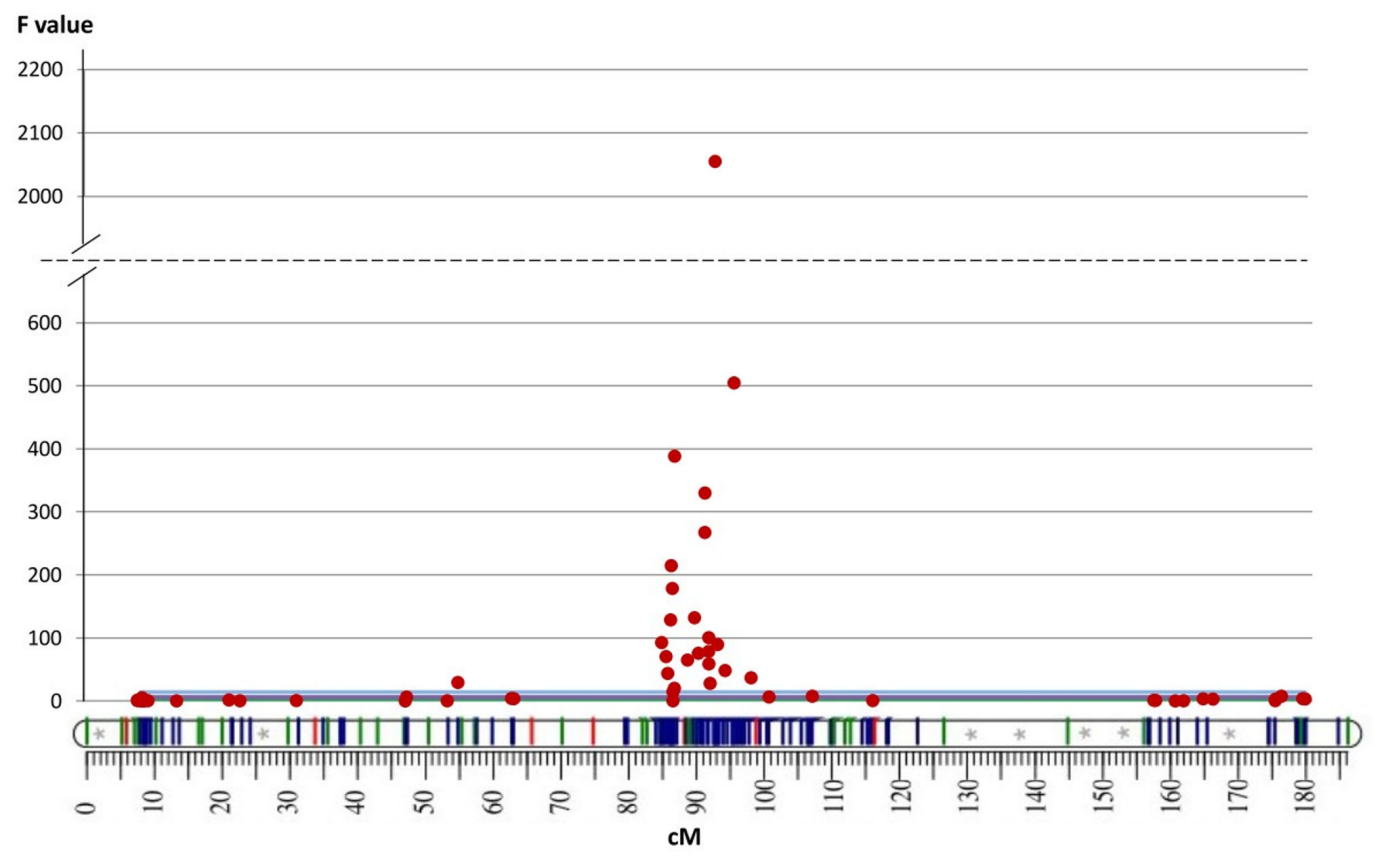
Pattern of F statistic from ANOVA along chromosome III **(the linkage group map under the F value graph is taken from the ‘Clementine’ genetic map [71]**. The blue line indicates the least significant value for F at p<0.01.

The pattern of this F parameter along the nine linkage groups of the ‘Clementine’ genetic map [71] led us to discard most genomic regions (Figure S1) and allowed identification of a region containing numerous markers with a high probability (>99%) of association with phenotype variation, located on chromosome III (Figure 4). This region includes 25 significant SNP markers within an interval of 13.1 cM between markers CiC4831-03 (at 84.66 cM) and CiC1875-01 (at 97.76 cM) on the ‘Clementine’ map. The maximum F value within this region is attained by marker CiC4681-02, located at 92.78 cM (F=2055). The genomic region between these two markers contains around 15 Mb. No significant marker clusters were found in any other area of the genome.

### Genetic mapping of the genomic region surrounding the ABS locus

Among the SNP markers with significant linkage to ABS resistance, five displayed the most convenient allelic conformation in the parents (heterozygous in ‘Fortune’ and homozygous in ‘Willowleaf’) for genetic mapping by individual genotyping of the ‘Fortune’ × ‘Willowleaf’ progeny. One SSR marker included in this segment in the ‘Clementine’ genetic map [71] also displayed useful allelic polymorphisms between parents.

To develop additional markers with useful allelic conformation, the genomic region (from www.phytozome.net) surrounding the 25 significant SNPs was scanned to find new microsatellites and develop new SSR markers. Among 42 SSRs tested, four new SSR markers provided useful polymorphisms. Moreover, 4.47 kb (Table S2) within this region in ‘Fortune’ and ‘Willowleaf’ and two SNPs heterozygous in ‘Fortune’ and homozygous in ‘Willowleaf’ were sequenced. More detailed information on all markers used in this study is available as supplementary material in Table S3 and Table S4.

Next, five mapped SNP markers [71], one mapped SSR marker [CX0038 [42]:], and six newly developed markers (four SSRs and two SNP markers) were used to genotype all 93 triploid hybrids of the ‘Fortune’ × ‘Willowleaf’ population. Because the male parent was homozygous or different from the female parent at each selected locus, the genetic structure of the diploid female gamete (Table S5) was deduced from the triploid hybrid genotyping [see 41 for details], and the marker HRs were estimated.

**Figure 5:**
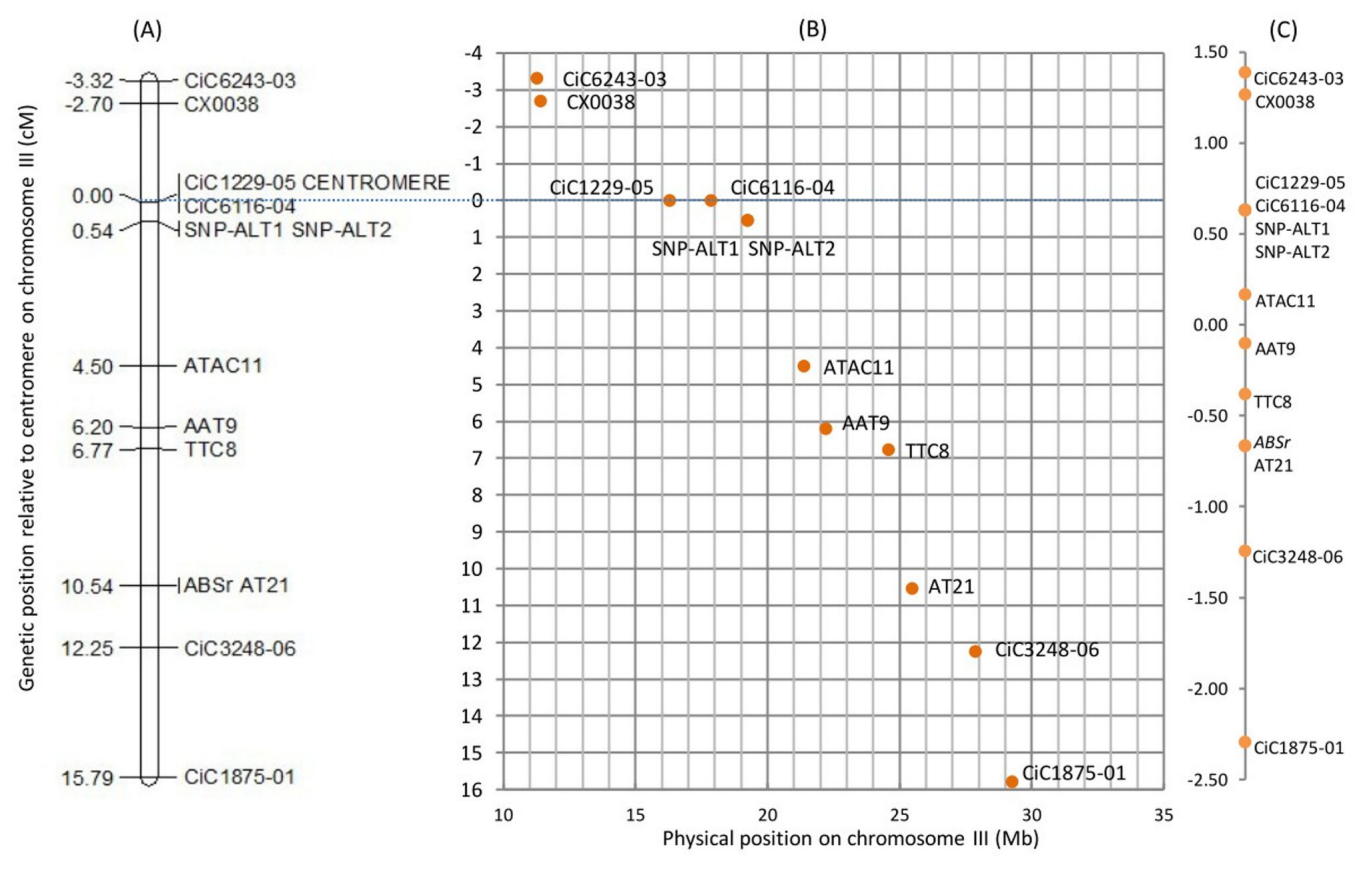
Order and location of markers and *ABSr* locus. (A) De novo genetic mapping (cM) of markers and the *ABSr* locus on chromosome III relative to the centromere by half-tetrad analysis, (B) relation between genetic and physical location in the ‘Clementine’ reference genome (www.phytozome.net/clementine), and (C) representation of the markers on the first axis of the multiple correspondence analysis.

We took advantage of the direct link between HR in 2*n* gametes and the locus-centromere distance for *de novo* mapping of genetic markers in relation to the centromere position, using the Cx(Co)^4^ model for SDR with partial interference [41]. No recombination was observed between the centromere and the CiC1229-05 and CiC6116-04 markers. The markers next closest to centromere were SNP-ALT1 and SNP-ALT2 (with the same HTA data), 0.54 cM away; the next closest marker was CX0038 (2.7 cM). To determine whether this marker was located at one side or the other of the centromere, we checked its correlation with the markers in distal positions on the draft map (at this step, CiC1229-05 and CiC6116-04 on one side, and SNP-ALT1 and SNP-ALT2 on the other side). Because the lower correlation was for SNP-ALT1 and SNP-ALT2, CX0038 was positioned on the opposite chromosome arm. The same process was applied at each subsequent step of marker addition to the map, proceeding according to increasing distance from the centromere. The order of the mapped markers in the *de novo* map was the same as in the ‘Clementine’ map [71], and new (non-mapped) markers maintained the expected order of the assembled sequence available at www.phytozome.net (Figure 5A,B). However, the estimated genetic distances were higher than those on the ‘Clementine’ map, suggesting that the recombination rate in this genomic region during the production of the 2*n* gamete was higher in ‘Fortune’ than in ‘Clementine’. A logically important modification of the slope of the physical distances according to genetic map is observed in the centromeric region (with lower recombination by physical distance unit).

No recombination was observed between the AT21 marker and the *ABSr* locus. The two flanking markers (TTC8 and CiC3248-06) were found at 3.77 and 1.71 cM, respectively, from the *ABSr* locus, delimiting a 3.3 Mb genome region. This position of the *ABSr* locus was checked by an MCA based on a qualitative matrix (see Material and Methods). Most of the matrix diversity was represented in the first axis (72.9%; Figure 5C), where the order of markers and the relative position of the *ABSr* locus was identical to the *de novo* mapping, based on HR.

### Gene annotations around the *ABSr* locus

The assembled sequence (www.phytozome.org) of the region of chromosome III between the two markers flanking the *ABSr* locus (TTC8 and CiC3248-06) was examined for gene annotations. The results revealed several disease resistance genes along and at the extremes of the analysed region, so the analysis was extended 1.5 Mb down from the TTC8 marker and 1.7 Mb up from the CiC3248-06 marker. Ninety-five genes annotated as homologous to disease resistance genes were found within the corresponding 6.5 Mb region. A genome-wide analysis of disease resistance gene homologs revealed that 17% of them are located within this region on chromosome III.

**Figure 6:**
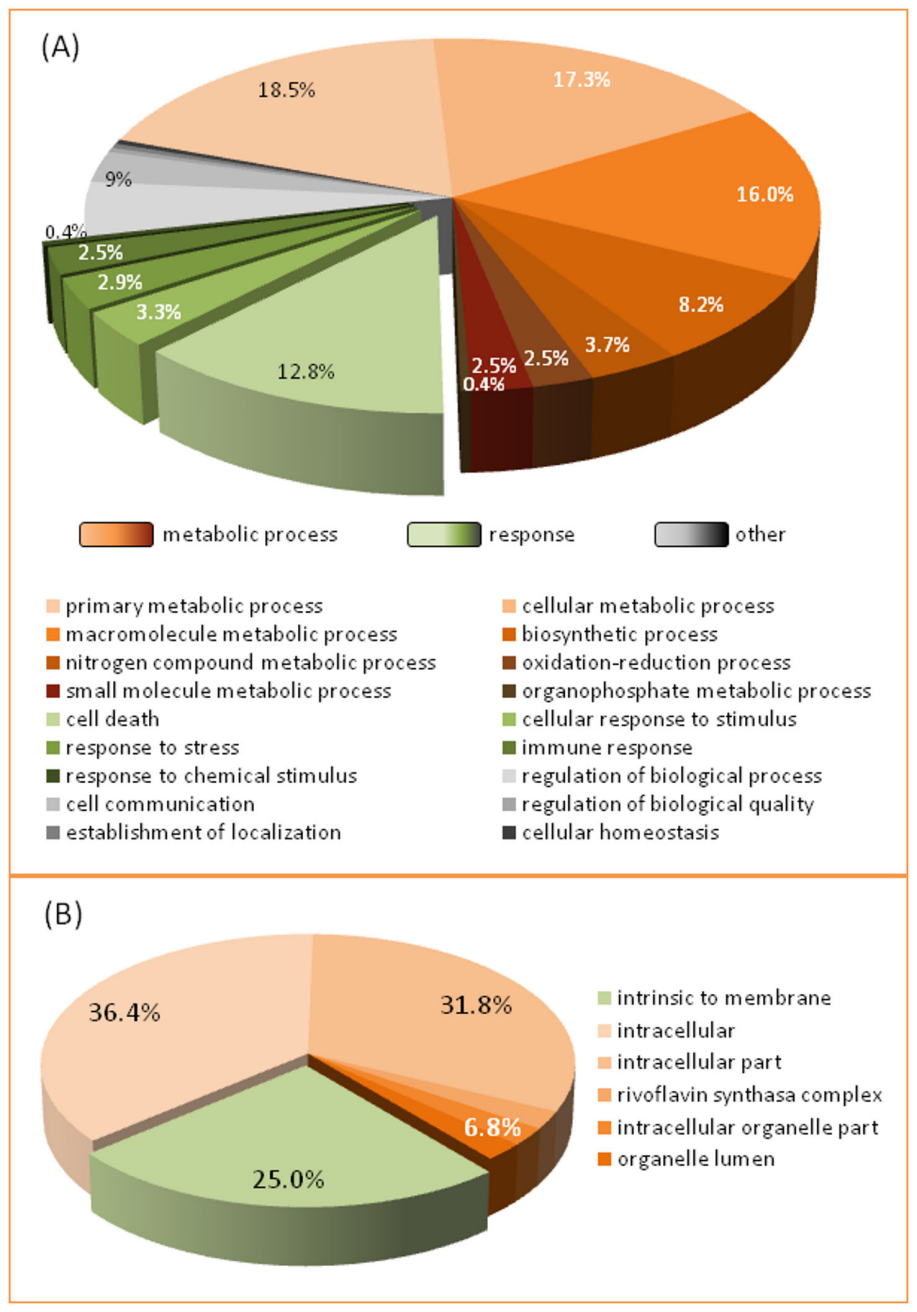
Classification of genes annotated between the TTC8 and CiC3248-06 markers according to gene ontology (GO) functional categories. (A) GO biological process categories. (B) GO cellular component categories.

Within the 3.3-Mb region defined by the two flanking markers, 177 annotated genes were found (Table S6). Gene ontology (GO) analysis of biological processes revealed that 69.1% of these genes are involved in metabolic processes and 21.9% are related to response or cell death (Figure 6A). GO also indicated that 25% of the genes are intrinsic to the membrane (Figure 6B), which is the target of the ACT-toxin produced by the tangerine pathotype of *A. alternata*.

**Figure 7:**
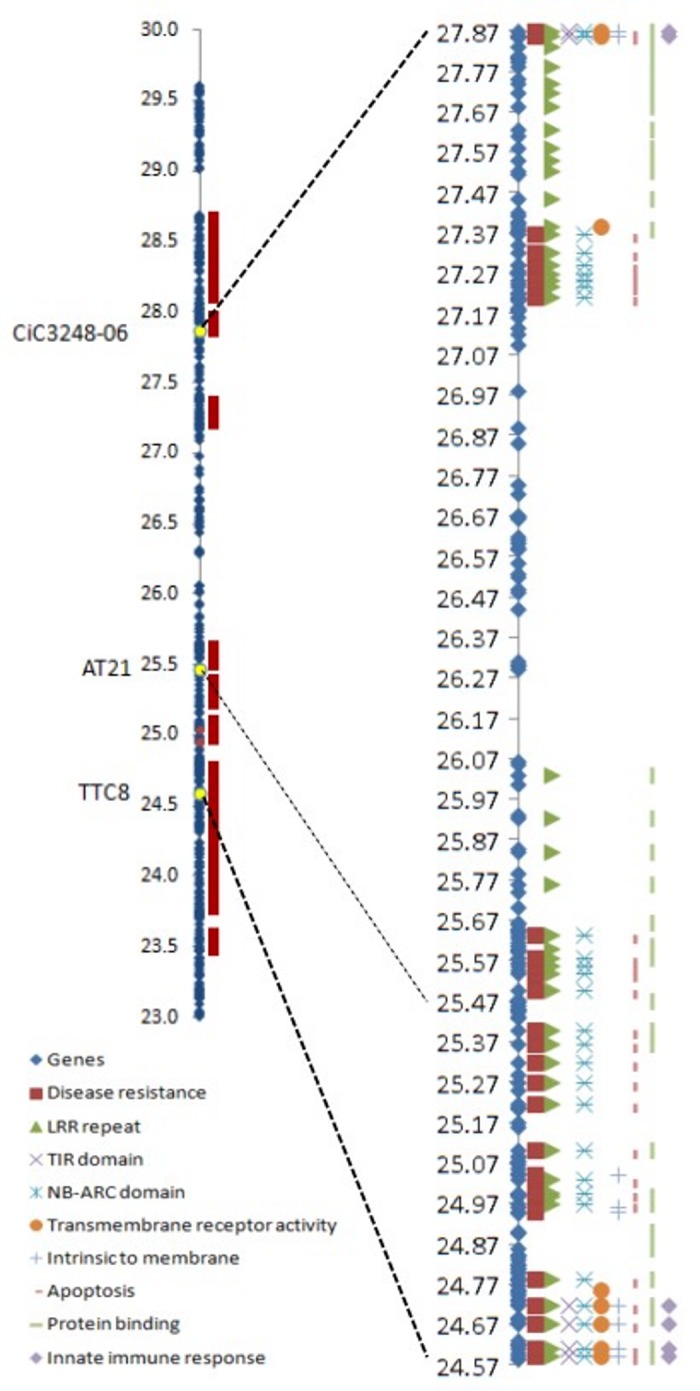
Genes found in the region around the *ABSr* locus, indicating the disease resistance gene homologous and their major domains and annotations.

In the region defined by the two flanking markers, which includes the *ABSr* locus, 33 disease resistance homologous genes were found (Figure 7). Thirty of these genes encode proteins predicted to have a central nucleotide-binding site (NBS) domain, 28 are involved in apoptosis, and 29 have a C-terminal leucine-rich repeat (LRR) domain. Six of the 30 NBS-containing genes have transmembrane activity. Among the resistance genes identified, 15 are homologous to the *LOV1* gene, which has been implicated in dominant susceptibility of *Arabidopsis* to the victorin toxin produced by *Cochliobolus victoriae* Nelson [80]. Other three of these resistance genes belong to the *mlo* family, which confer durable broad-spectrum resistance against the powdery mildew pathogen in barley [81]. 

## Discussion

### The monolocus inheritance and recessivity of ABS resistance was confirmed in citrus triploid progeny, and the *ABSr* locus was mapped in the chromosome III genetically close to the centromere

Several studies have reported the single dominance inheritance of ABS susceptibility in diploid citrus genotypes [19,22]; however, no data have been previously published regarding triploid progeny. In this present study, inheritance of resistance to the ABS pathogen has been analysed in triploid progeny produced by different strategies (sexual polyploidisation and interploid crosses) over a wide range of genetic backgrounds. The segregations (resistance/susceptibility) observed for all the triploid populations we evaluated confirm the monolocus inheritance and recessivity of the disease in a triploid context. All progeny arising from a homozygous susceptible cultivar, such as ‘Minneola’, were susceptible, whereas in cases of heterozygous parents, segregations were as expected, depending on the hybridisation strategy.

Genetic mapping and marker-trait association in polyploids are complicated by the diversity of the meiotic process (and therefore, recombination mechanisms) involved, as well as the distribution of markers into multiple dosage classes [82]. Despite these limitations, genetic maps based on segregating molecular markers have been generated for a wide range of polyploids including tetraploid cotton [83], hexaploid tall fescue [84], hexaploid wheat [85] and octoploid sugar-cane mandarin [82,86].

In this study, the first information regarding the location of the *ABSr* locus was given directly by the analysis of the segregation between susceptible and resistant triploid hybrids in progeny obtained by sexual polyploidisation (2*n* gametes), assuming monolocus recessive determination of the resistance. Indeed, for 2*n* gametes resulting from either FDR or SDR, as in the ‘Fortune’ mandarin [41], there is a direct linkage between parental HR (and therefore, the proportion of resistant and susceptible hybrids) and the genetic distance to the centromere. HTA is therefore an efficient way to map loci relative to a centromere [78,87,88]. At this step, we identified that the *ABSr* locus was relatively close (10.5 cM) to one centromere.

The *ABSr* locus was then located by combining two approaches. The first one was to perform BSA coupled with genome-scan using SNP markers mapped in the reference ‘Clementine’ genetic map [71]. This approach allowed localisation of the ABS resistance locus within a 13.1 cM area on chromosome III of the ‘Clementine’ genetic map, corresponding to 15 Mb of scaffold 3 of the current ‘Clementine’ whole genome assembly in pseudomolecules (www.phytozome.net). Our results confirm the potential for raw location of major genes involved in phenotypic trait variability by coupling BSA strategies with genomic scanning, as previously proposed by [57], and demonstrate that it can be successfully applied in a polyploid segregating population.

This genomic region was further examined for additional SSR and SNP markers, which were used for de novo mapping of the area by HTA of 2*n* gamete inferred from triploid hybrid genotypes. Mapping functions relating HR and centromere distance [41,78] and an approach based on a correlation matrix have given convergent results in identification of the closest markers flanking the candidate ABS resistance gene. The AT21 marker appeared to be tightly linked to the *ABSr* locus. However, it would be necessary to analyse many more progeny to estimate its linkage with the *ABSr* locus precisely. The two flanking markers of AT21 and the *ABSr* locus are TTC8 and CiC3248-06, respectively, at 3.77 and 1.71 cM. This marker frame is much more closely linked than previous markers identified from diploid segregating progenies. Two RAPD markers are in loose linkage with the *ABSr* locus (15.3 cM and 36.7 cM far from the ABS locus in the same side) [19]. A more recent study identified two flanking SRAP markers at 3 cM and 13 cM, and the authors of that study proposed that the genomic region of the *ABSr* locus should display low polymorphism, explaining the difficulty of obtaining markers very close to the gene [22]. The availability of the reference genetic map [71] and whole genome sequence [72] of *C. clementina* clearly increases the potential for marker-trait association studies in citrus, with co-dominant markers located both in the physical and genetic maps.

### Candidate genes for resistance to ABS were identified

The *ABSr* locus appears to be included in a genomic region very rich in disease resistance gene homologs. Indeed, 17% of all resistance genes annotated in the citrus reference genome (www.phytozome.net) are found in a 6.5 Mb region (2.2% of the whole genome) of chromosome III, surrounding the *ABSr* locus. In the 3.3 Mb region defined by the two flanking markers, 33 disease resistance gene homologs were identified. Six of them are considered to be intrinsic to the membrane, including three belonging to the *mlo* family and 28 related to apoptosis. These resistance genes are organised in clusters, as generally described in many crop species [89,90], and as already demonstrated in citrus for resistance to Tristeza virus found in the *Poncirus* genome [91].

*A. alternata* is a necrotroph pathogen, which first kills host cells before parasitizing them and metabolising their contents. If the toxins used to kill host cells are not released at the right time, place, or concentration, or if a particular host genotype is insensitive to the toxin, the host cells will not die, the necrotroph will be unable to infect or reproduce, and the plant will be resistant [92]. *A. alternata*, like other necrotrophs, produces host-selective toxins (ACT-toxins), defined as pathogen effectors, which induce toxicity and promote disease only in the host species expressing a specific and often dominant susceptibility gene [93]. Their pathogenic ability is conditioned by a gene in the pathogen that encodes production of the toxin and by a gene in the host that promotes sensitivity to the toxin. For this type of pathogen, plant resistance can be achieved via the loss or modification of the toxin’s target or through detoxification [94].

Inheritance of ABS resistance in citrus has been described as monogenic [19,22], controlled by a single recessive allele. The results of this study corroborate this hypothesis by demonstrating the predicted proportions of resistant and susceptible genotypes obtained from various crosses and the identification, by BSA, of a single genomic region highly associated with resistance. ACT-toxins from the tangerine pathotype of *A. alternata*, as well as AF-toxin from the strawberry pathotype and AK-toxin from the Japanese pear pathotype, have an epoxy-decatrienoic acid structure and exert their primary effect on the plasma membrane of susceptible cells, causing a rapid increase in electrolyte loss from tissues and invaginations in plasma membranes [95]. Varietal resistance to ACT-toxin in citrus is very highly correlated with ABS resistance. Therefore, a probable function for the gene of interest is to encode a protein involved in ACT-toxin recognition, which would allow the toxin to cause cell death. Such a dominant gene should be present (homozygously or heterozygously) in susceptible cultivars, and absent or defective in resistant cultivars.

The most obvious candidate for providing recognition specificity to the pathogen effector is the LRR domain, which binds a corresponding ligand [94] with a putative nucleotide-binding (NB) site; these genes are classified as ‘NB-LRR’ genes [96]. This class includes members that carry either N-terminal homology to the Toll protein and interleukin-1 receptor (TIR-NB-LRR) or a putative coiled-coil (CC) at the N-terminus (CC-NB-LRR). Resistance (R) genes from both of these subclasses confer resistance against fungi, and several fungal resistance genes have been reported and used in crop improvement programs. NB-LRR genes have been identified that confer resistance against flax rust, maize rust, barley powdery mildew, rice blast and *Fusarium* wilt and downy mildew of tomato [97]. However, sequence variation within the central LRR domain, as well as variation in LRR copy number, plays an important role in determining recognition speciﬁcity [98]. Likewise, *R* genes, first identified as dominant resistance genes, could be targets of pathogen effectors and therefore play roles in susceptibility [99]. Thus, avirulence (Avr) elicitors and HST may be recognising the same resistance genes in plants, leading to evolutionary outcomes that differ between necrotrophs and biotrophs while affecting the evolution of the corresponding R genes [100]. In *Arabidopsis*, victorin (an HST produced by *C. victoriae*) sensitivity and disease susceptibility is conferred by the *LOV1* gene, which encodes a NB-LRR protein. *LOV1* is targeted by victorin, the pathogen effector, and this interaction results in disease susceptibility [80]. These NB-LRR proteins recognise specific pathogen-derived products and initiate a resistance response that often includes a type of cell death known as the hypersensitive response [101]. In the same way, the *Pc* locus of sorghum, which contains genes encoding NB-LRR proteins, determines dominant susceptibility to HSTs produced by the necrotroph fungus *Periconia circinata* (L). Mangin Sacc [102,103]. . Together, these results suggest that for necrotroph fungi, the disease is favoured by inducing the resistance response [95,99]; this mode of susceptibility could also apply to *A. alternata*. In this study, thirty disease resistance gene homologous encoding proteins with NBSs were found in the ABS locus region, and 15 of them are homologous to the *LOV1* gene. Therefore, disease resistance gene homologous should be considered as candidate genes for inducing susceptibility, especially in the case of *LOV1* homologs found in this region.

Another class of resistance genes, belonging to the *mlo* family, has also been implicated in susceptibility to barley powdery mildew produced by *Blumeria graminis* f. sp. *hordei* [81]. Mlo proteins are localised in the plasma membrane and possess seven transmembrane domains; it has been suggested that they function as receptors in plants [81,104,105]. The resistance trait conferred by *mlo* is recessively inherited and non-race-specific, because it is effective against all isolates of the fungus *B. graminis* [106,107]. Three resistance genes found in the *ABSr* locus region belong to this class. However, in citrus, two pathotypes of *A. alternata* have been described that produce HSTs that affect a narrow range of genotypes (ACT-toxin to tangerines, ACR-toxin to Rough lemon [*C. jambhiri* Lush.] and Rangpur lime [*C. limoniae* Osbeck]), and resistance found in the germplasm was pathotype-specific [4].

The identification of the gene for ABS resistance will involve fine mapping with large diploid populations. SNP markers are currently being developed from each candidate gene for this purpose. From the reduced set of candidate genes that would result from this fine genetic mapping, functional validation could be performed by genetic transformation [108] or viral vector-induced gene silencing [109,110].

For susceptible genotypes it is probable that additional genes, but also environmental factors, affect the susceptibility level. QTLs analyses conducted in susceptible progeny should be necessary to decipher this quantitative component of susceptibility.

### Toward efficient breeding for ABS resistance

ABS is a major fungal disease in certain mandarin cultivars around the world; the disease causes a substantial loss of production and fruit quality [1,4]. Currently, ABS management relies mainly on the application of fungicides [12-14], but this control is expensive, not environmentally friendly, and not always efficient. As a consequence, the production of susceptible cultivars, such as 'Fortune' and ‘Nova’ among others, has declined significantly during recent years, and many trees of the most susceptible varieties have been removed and replaced by resistant cultivars that may lack some of the interesting agronomic traits of the susceptible cultivars [111]. Therefore, ABS resistance must be considered as a major selection criterion in mandarin breeding programs.

Our results demonstrate that it is possible to use susceptible parents heterozygous for the resistance gene to breed resistant triploid varieties. For instance, the susceptible cultivar ‘Fortune’, which is a very efficient female parent in producing high-quality triploid hybrids in 2*x* × 2*x* hybridisation [28], should not be discarded. Indeed, the 39% and 19% of resistant triploid hybrids produced when crossed with resistant or heterozygous susceptible genotypes, respectively, are acceptable if combined with early selection by controlled inoculation phenotyping or MAS. On the other hand, parents homozygous for the susceptible allele, such as ‘Minneola’, should be definitively ruled out. Our results also demonstrate that when heterozygous susceptible parents are used as producers of diploid gametes, it is much more efficient to integrate them in a 2*x* × 2*x* strategy rather than to use them as doubled-diploid parents in interploid crosses. Indeed, the heterozygosity transmission of the *ABSr* locus (associated with susceptibility transmission to the triploid progeny) is lower in the 2*n* gametes than in the diploid gametes produced by doubled-diploids, due to its location close to the centromere of chromosome III and the SDR origin of unreduced gamete formation in most citrus genotypes [43].

HTA has permitted identification two flanking markers at 3.77 and 1.71 cM of the *ABSr* locus, as well as a third marker that did not exhibit any recombination with the *ABSr* locus within the analysed population. These markers should be used together for efficient early MAS for different parental combinations when the markers are heterozygous in the susceptible parent and polymorphic between the two parents. We are currently sequencing DNA fragments between these two markers to identify SNP loci that provide a useful allelic combination for the various crosses of our mandarin breeding program. These are examples of the very few identified markers for MAS in citrus, which include the SSR markers flanking the Citrus Tristeza virus resistance gene(s) of *Poncirus* [ [112]; Mikeal Roose, personal communication] and the dominant PCR assay for the anthocyanin content of pulp of blood orange due to a transposable element in the 5’ extremity of the Ruby gene [113].

## Supporting Information

Table S1 **Number of individuals (N) evaluated within each population, and percentage of hybrids evaluated as resistant (%R).**
(DOCX)

Table S2 **Primers used to sequence the 4.47 kb genomic region on scaffold 3 (www.phytozome.net/clementine) surrounding the SNPs identified by BSA-genome scan as linked to ABS resistance.**
(DOCX)

Table S3 **Information about SSR and SNP markers used in this study, indicating the alleles in the parental lines and the expected genotypes within the ‘Fortune’ (F) × ‘Willowleaf’ (WL) triploid progeny.**
(DOCX)

Table S4 **Information about new SSR and SNP markers developed.**
(DOCX)

Table S5 **Allelic configuration for the analyzed markers of 93 diploid female gametes within the ‘Fortune’ × ‘Willowleaf’ population.**
(DOCX)

Table S6 **Annotations between 24.57 Mb (TTC8 marker) and 27.87 Mb (CiC3248-06 marker) in scaffold 3 (www.phytozome.net).**
(DOCX)

Figure S1 **Values of F parameter from ANOVA along the nine linkage groups of the ‘Clementine’ genetic map [71].**
(DOCX)
