# Supplementary material for: Genetically Based Location from Triploid Populations and Gene Ontology of a 3.3-Mb Genome Region Linked to Alternaria Brown Spot Resistance in Citrus Reveal Clusters of Resistance Genes
Source: PLoS One. 2013 Oct 8;8(10):e76755. doi: 10.1371/journal.pone.0076755 (PMC3792864; doi:10.1371/journal.pone.0076755)
Supplement: Table S1 — Number of individuals (N) evaluated within each population, and percentage of hybrids evaluated as resistant (%R). (DOCX) [file pone.0076755.s001.docx]

**Table S1. Number of individuals (N) evaluated within each population, and percentage of hybrids evaluated as resistant (%R)**

|  | **Population** | | | | |
| --- | --- | --- | --- | --- | --- |
|  | **Orri X Oronules** | **Clemenules X Nova** | **Fortune X Scarlett** | **Fina X Fairchild 4x** | **Fortune X Kara 4x** |
| **Segregation** | <aa X aa> | <aa X Aa> | <Aa X aa> | <aa X AAaa> | <Aa X aaaa> |
| **N** | 16 | 11 | 47 | 15 | 25 |
| **% R** | 100% | 54.55% | 38.3% | 20% | 52% |
