# Supplementary material for: Genetically Based Location from Triploid Populations and Gene Ontology of a 3.3-Mb Genome Region Linked to Alternaria Brown Spot Resistance in Citrus Reveal Clusters of Resistance Genes
Source: PLoS One. 2013 Oct 8;8(10):e76755. doi: 10.1371/journal.pone.0076755 (PMC3792864; doi:10.1371/journal.pone.0076755)
Supplement: Table S2 — Primers used to sequence the 4.47 kb genomic region on scaffold 3 (www.phytozome.net/clementine) surrounding the SNPs identified by BSA-genome scan as linked to ABS resistance. (DOCX) [file pone.0076755.s002.docx]

**Table S2. Primers used to sequence the 4.47 kb genomic region on scaffold 3 (http:://www.phytozome.net/clementine) surrounding the SNPs identified by BSA-genome scan as linked to ABS resistance**

| **Primer Forward** | **Primer Reverse** | **Product size** | **Position in scaffold 3 (bp)** |
| --- | --- | --- | --- |
| CAATTTGAGCTCGCTTATTT | GGTTCATCTAGGTCACCTTCT | 1154 | 19240437 to 19241590 |
| TAAAACTTGGCATGGATCTT | CATATGGAATCTTCCCAGTC | 1176 | 19241527 to 19242702 |
| TGCCAGCTATGATAAGAACA | AGACAAAATTATCCCACTGTGT | 1170 | 19242617 to 19243786 |
| ATTTAAATGATGAATTTGATGC | TTATCTTTGCTGCATTTGAA | 1175 | 19243732 to 19244906 |

Positions from http:://www.phytozome.net/clementine
