# Supplementary material for: Genetically Based Location from Triploid Populations and Gene Ontology of a 3.3-Mb Genome Region Linked to Alternaria Brown Spot Resistance in Citrus Reveal Clusters of Resistance Genes
Source: PLoS One. 2013 Oct 8;8(10):e76755. doi: 10.1371/journal.pone.0076755 (PMC3792864; doi:10.1371/journal.pone.0076755)
Supplement: Table S3 — Information about SSR and SNP markers used in this study, indicating the alleles in the parental lines and the expected genotypes within the ‘Fortune’ (F) × ‘Willowleaf’ (WL) triploid progeny. (DOCX) [file pone.0076755.s003.docx]

**Table S3. Information about SSR and SNP markers used in this study, indicating the alleles in the parental lines and the expected genotypes within the ‘Fortune’ (F) × ‘Willowleaf’ (WL) triploid progeny**

| **Marker id** | **Marker type** | **F alleles** | **WL alleles** | **Expected 3x genotypes** | **Reference** |
| --- | --- | --- | --- | --- | --- |
| CiC1229-05 | SNP (T/G) | TG | TT | TTT, TTG, TGG | Ollitrault et al., 2012a |
| CiC1875-01 | SNP (T/C) | TC | CC | CCC, TCC, TTC | Ollitrault et al., 2012a |
| CiC3248-06 | SNP (A/G) | AG | AA | AAA, AAG, AGG | Ollitrault et al., 2012a |
| CiC6116-04 | SNP (T/C) | TC | CC | CCC, TCC, TTC | Ollitrault et al., 2012a |
| CiC6243-03 | SNP (A/G) | AG | GG | GGG, AGG, AAG | Ollitrault et al., 2012a |
| CX0038 | SSR | 337/339 | 339/339 | 319/319/321, 319/321/321, 321/321/321 | Chen et al., 2008 |
| ATAC11 | SSR | 248/264 | 256/264 | 248/248/256, 248/248/264, 248/256/264, 248/264/264, 256/264/264, 264/264/264 | new |
| AAT9 | SSR | 260/266 | 266/269 | 260/260/266, 260/260/269, 260/266/266, 260/266/269, 266/266/266, 266/266/269 | new |
| TTC8 | SSR | 197/203 | 203/null | 197/197/203, 197/197/null, 197/203/203, 197/203/null, 203/203/203, 203/203/null | new |
| AT21 | SSR | 193/205 | 177/205 | 177/193/193, 193/193/205, 177/193/205, 193/205/205, 177/205/205, 205/205/205 | new |
| SNPALT1-Y | SNP (C/T) | CT | CC | CCC, CCT, CTT | new |
| SNPALT2-K | SNP (G/T) | GT | TT | GGT, GTT, GGT | new |
