# Supplementary material for: Genetically Based Location from Triploid Populations and Gene Ontology of a 3.3-Mb Genome Region Linked to Alternaria Brown Spot Resistance in Citrus Reveal Clusters of Resistance Genes
Source: PLoS One. 2013 Oct 8;8(10):e76755. doi: 10.1371/journal.pone.0076755 (PMC3792864; doi:10.1371/journal.pone.0076755)
Supplement: Table S4 — Information about new SSR and SNP markers developed. (DOCX) [file pone.0076755.s004.docx]

**Table S4. Information about new SSR and SNP markers developed**

| **Marker id** | **Position in scaffold 3 (bp)** | **Allele polymorphism or repeat motif** | **Sequence flanking the SNP** | **Forward primer** | **Reverse primer** | **Melting Temp (ºC)** |
| --- | --- | --- | --- | --- | --- | --- |
| SNPALT1-Y | 19241709 | C/T | CCAGACTCGTCACCACCACGCCTCCTTCCATCCAAATCGGCTGCACCTAATGTTGATGATACCACGCTGGCT[C/T]TAACTGTTGCCCAAGCCCGCCAAACCCAATCTAGGCCCATTGACCCCAGCCAACAC |  |  |  |
| SNPALT2-K | 19243610 | G/T | GTATATGAATTTTTTTAGTTTTACGATGGATCTATT[G/T]TCACGTGAAAATTGGTTTCCAATTGCCTTCGCCTCACATACCATATG |  |  |  |
| ATAC11 | 21375818 to 21376080 | ATAC |  | GTCGGATTCCTCTATCAACA | TCAAGCAAGCATTTCAATAA | 55 |
| AAT9 | 22207742 to 22208005 | AAT |  | TTACTTCACCTCCCTGAAAA | CAAGAATTGGGACAACTGAT | 55 |
| TTC8 | 24579024 to 24579224 | TTC |  | TACATATCAAGCGCACAGAC | GACAGAGCCGAATAGAGATG | 55 |
| AT21 | 25473601 to 25473785 | AT |  | TAAAATTCTGCACCGATGA | GGCTTCATTTTATTGCTTGT | 55 |

Positions from http:://www.phytozome.net/clementine
