# Supplementary material for: Genetically Based Location from Triploid Populations and Gene Ontology of a 3.3-Mb Genome Region Linked to Alternaria Brown Spot Resistance in Citrus Reveal Clusters of Resistance Genes
Source: PLoS One. 2013 Oct 8;8(10):e76755. doi: 10.1371/journal.pone.0076755 (PMC3792864; doi:10.1371/journal.pone.0076755)
Supplement: Table S5 — Allelic configuration for the analyzed markers of 93 diploid female gametes within the ‘Fortune’ × ‘Willowleaf’ population. (DOCX) [file pone.0076755.s005.docx]

**Table S5. Allelic configuration for the analyzed markers of 93 diploid female gametes within the ‘Fortune’ × ‘Willowleaf’ population**

|  | **CiC6243-03** | **CX0038** | **CiC1229-05** | **CiC6116-04** | **SNP-ALT1** | **SNP-ALT2** | **ATAC11** | **AAT9** | **TTC8** | **AT21** | **CiC3248-06** | **CiC1875-01** |  | ***ABSr*** |
| --- | --- | --- | --- | --- | --- | --- | --- | --- | --- | --- | --- | --- | --- | --- |
|  | aa | aa | aa | aa | aa | aa | aa | aa | aa | aa | aa | aa |  | a- |
|  | aa | aa | aa | aa | aa | aa | aa | aa | aa | aa | aa | aa |  | a- |
|  | aa | aa | aa | aa | aa | aa | aa | aa | aa | aa | aa | aa |  | a- |
|  | bb | bb | bb | bb | bb | bb | bb | bb | bb | bb | ab | ab |  | bb |
|  | ab | ab | bb | bb | bb | bb | bb | bb | bb | bb | bb | bb |  | bb |
|  | aa | aa | aa | aa | aa | aa | aa | aa | aa | aa | aa | aa |  | a- |
|  | aa | aa | aa | aa | aa | aa | aa | aa | aa | aa | aa | aa |  | a- |
|  | bb | bb | bb | bb | bb | bb | bb | bb | bb | bb | bb | bb |  | bb |
|  | aa | aa | aa | aa | aa | aa | aa | aa | ab | ab | aa | ab |  | a- |
|  | bb | bb | bb | bb | bb | bb | bb | bb | bb | bb | bb | bb |  | bb |
|  | bb | bb | bb | bb | bb | bb | bb | bb | bb | bb | bb | bb |  | bb |
|  | bb | bb | bb | bb | bb | bb | ab | ab | bb | bb | bb | bb |  | bb |
|  | aa | aa | aa | aa | aa | aa | aa | aa | aa | aa | aa | aa |  | a- |
|  | aa | aa | aa | aa | aa | aa | aa | aa | aa | aa | aa | aa |  | a- |
|  | aa | aa | aa | aa | aa | aa | aa | aa | aa | ab | ab | ab |  | a- |
|  | aa | aa | aa | aa | aa | aa | aa | aa | aa | aa | aa | aa |  | a- |
|  | bb | bb | bb | bb | bb | bb | bb | bb | bb | bb | bb | bb |  | bb |
|  | bb | bb | bb | bb | bb | bb | bb | bb | bb | bb | bb | bb |  | bb |
|  | bb | bb | bb | bb | bb | bb | ab | ab | aa | aa | ab | ab |  | a- |
|  | aa | aa | aa | aa | aa | aa | ab | ab | aa | ab | aa | aa |  | a- |
|  | aa | aa | aa | aa | aa | aa | aa | aa | ab | ab | ab | ab |  | a- |
|  | aa | aa | aa | aa | aa | aa | aa | aa | aa | aa | aa | aa |  | a- |
|  | bb | bb | bb | bb | bb | bb | bb | bb | bb | bb | bb | bb |  | bb |
|  | aa | aa | aa | aa | aa | aa | aa | aa | aa | aa | ab | ab |  | a- |
|  | aa | aa | aa | aa | aa | aa | aa | aa | aa | aa | aa | ab |  | a- |
|  | bb | bb | bb | bb | bb | bb | aa | aa | aa | aa | bb | ab |  | a- |
|  | bb | bb | bb | bb | bb | bb | bb | bb | bb | bb | bb | bb |  | bb |
|  | bb | bb | bb | bb | bb | bb | bb | bb | bb | bb | ab | ab |  | bb |
|  | bb | bb | bb | bb | bb | bb | bb | bb | bb | bb | bb | bb |  | bb |
|  | bb | bb | bb | bb | bb | bb | bb | bb | bb | bb | bb | ab |  | bb |
|  | aa | aa | aa | aa | aa | aa | aa | aa | aa | aa | aa | aa |  | a- |
|  | aa | aa | aa | aa | aa | aa | aa | aa | aa | aa | aa | aa |  | a- |
|  | aa | aa | aa | aa | aa | aa | aa | aa | aa | aa | aa | aa |  | a- |
|  | aa | aa | aa | aa | aa | aa | aa | aa | aa | ab | ab | ab |  | a- |
|  | aa | aa | aa | aa | aa | aa | aa | aa | aa | aa | aa | aa |  | a- |
|  | aa | aa | aa | aa | aa | aa | aa | aa | aa | aa | aa | aa |  | a- |
|  | bb | bb | bb | bb | bb | bb | bb | bb | bb | bb | bb | bb |  | bb |
|  | bb | bb | bb | bb | bb | bb | bb | bb | bb | bb | bb | bb |  | bb |
|  | aa | aa | aa | aa | aa | aa | aa | aa | aa | aa | aa | ab |  | a- |
|  | aa | aa | aa | aa | aa | aa | aa | aa | aa | aa | aa | aa |  | a- |
|  | aa | aa | aa | aa | aa | aa | aa | aa | aa | aa | aa | aa |  | a- |
|  | aa | aa | aa | aa | aa | aa | ab | ab | ab | ab | ab | ab |  | a- |
|  | bb | bb | bb | bb | bb | bb | ab | ab | ab | ab | ab | ab |  | a- |
|  | bb | bb | bb | bb | bb | bb | bb | bb | bb | bb | bb | bb |  | bb |
|  | bb | bb | bb | bb | bb | bb | bb | bb | bb | bb | bb | bb |  | bb |
|  | bb | bb | bb | bb | bb | bb | bb | bb | bb | bb | bb | bb |  | bb |
|  | aa | aa | aa | aa | aa | aa | aa | aa | ab | ab | ab | ab |  | a- |
|  | aa | aa | aa | aa | aa | aa | aa | aa | aa | aa | aa | aa |  | a- |
|  | aa | aa | aa | aa | aa | aa | aa | aa | aa | aa | aa | aa |  | a- |
|  | aa | aa | aa | aa | aa | aa | aa | aa | aa | ab | ab | ab |  | a- |
|  | bb | bb | bb | bb | bb | bb | bb | bb | bb | bb | bb | bb |  | bb |
|  | aa | aa | aa | aa | aa | aa | aa | aa | aa | aa | aa | aa |  | a- |
|  | aa | aa | aa | aa | aa | aa | aa | aa | aa | aa | aa | aa |  | a- |
|  | bb | bb | bb | bb | bb | bb | bb | bb | ab | ab | ab | ab |  | a- |
|  | aa | aa | aa | aa | aa | aa | aa | aa | aa | aa | ab | ab |  | a- |
|  | aa | aa | aa | aa | aa | aa | aa | aa | aa | aa | aa | aa |  | a- |
|  | bb | bb | bb | bb | bb | bb | bb | bb | bb | bb | bb | bb |  | bb |
|  | bb | bb | bb | bb | bb | bb | bb | bb | bb | bb | bb | ab |  | bb |
|  | ab | ab | bb | bb | bb | bb | bb | bb | bb | bb | bb | bb |  | bb |
|  | bb | bb | bb | bb | bb | bb | bb | bb | bb | bb | bb | bb |  | bb |
|  | aa | aa | aa | aa | aa | aa | aa | aa | aa | aa | aa | aa |  | a- |
|  | aa | aa | aa | aa | aa | aa | aa | aa | aa | aa | aa | aa |  | a- |
|  | bb | bb | bb | bb | bb | bb | bb | bb | bb | bb | ab | ab |  | bb |
|  | aa | aa | aa | aa | aa | aa | aa | aa | ab | ab | aa | aa |  | a- |
|  | ab | ab | bb | bb | bb | bb | bb | bb | bb | bb | bb | bb |  | bb |
|  | bb | bb | bb | bb | bb | bb | bb | bb | bb | ab | ab | ab |  | a- |
|  | ab | ab | bb | bb | bb | bb | bb | bb | bb | bb | bb | bb |  | bb |
|  | aa | aa | aa | aa | aa | aa | aa | aa | aa | aa | aa | aa |  | a- |
|  | bb | bb | bb | bb | bb | bb | bb | bb | bb | bb | bb | bb |  | bb |
|  | bb | bb | bb | bb | bb | bb | bb | ab | ab | ab | ab | aa |  | a- |
|  | ab | ab | bb | bb | bb | bb | bb | bb | bb | bb | bb | bb |  | bb |
|  | aa | aa | aa | aa | aa | aa | aa | aa | aa | aa | aa | ab |  | a- |
|  | bb | bb | bb | bb | bb | bb | bb | bb | bb | bb | bb | bb |  | bb |
|  | bb | bb | bb | bb | bb | bb | bb | bb | bb | bb | bb | bb |  | bb |
|  | aa | aa | aa | aa | aa | aa | aa | ab | ab | ab | ab | ab |  | a- |
|  | bb | bb | bb | bb | bb | bb | bb | bb | bb | ab | ab | ab |  | a- |
|  | aa | aa | aa | aa | ab | ab | ab | ab | ab | ab | ab | ab |  | a- |
|  | aa | aa | aa | aa | aa | aa | aa | aa | aa | aa | aa | aa |  | a- |
|  | ab | bb | bb | bb | bb | bb | bb | bb | bb | bb | bb | bb |  | bb |
|  | bb | bb | bb | bb | bb | bb | bb | bb | bb | bb | bb | bb |  | bb |
|  | aa | aa | aa | aa | aa | aa | aa | aa | aa | aa | aa | aa |  | a- |
|  | aa | aa | aa | aa | aa | aa | aa | aa | aa | aa | aa | aa |  | a- |
|  | bb | bb | bb | bb | bb | bb | bb | bb | bb | bb | bb | bb |  | bb |
|  | bb | bb | bb | bb | bb | bb | bb | bb | bb | bb | bb | bb |  | bb |
|  | bb | bb | bb | bb | bb | bb | bb | bb | bb | bb | bb | bb |  | bb |
|  | aa | aa | aa | aa | aa | aa | ab | ab | aa | aa | aa | aa |  | a- |
|  | bb | bb | bb | bb | bb | bb | bb | bb | bb | bb | bb | ab |  | bb |
|  | bb | bb | bb | bb | bb | bb | bb | bb | bb | bb | bb | bb |  | bb |
|  | aa | aa | aa | aa | aa | aa | aa | aa | aa | aa | aa | aa |  | a- |
|  | bb | bb | bb | bb | bb | bb | bb | bb | aa | ab | ab | ab |  | a- |
|  | bb | bb | bb | bb | bb | bb | bb | ab | ab | ab | ab | ab |  | a- |
|  | bb | bb | bb | bb | bb | bb | ab | ab | ab | ab | ab | ab |  | a- |
|  | aa | aa | aa | aa | aa | aa | aa | aa | aa | aa | aa | aa |  | a- |
| %HR (ab) | 6.45% | 5.38% | 0.00% | 0.00% | 1.08% | 1.08% | 8.60% | 11.83% | 12.90% | 20.43% | 23.91% | 31.11% |  | 20.43% |
| Physical position (Mb) | 11.26 | 11.40 | 16.29 | 17.86 | 19.24 | 19.24 | 21.38 | 22.21 | 24.57 | 25.47 | 27.87 | 29.26 |  | - |

**%**HR: percentage of maternal heterozygosity restitution
