# Supplementary material for: Genetically Based Location from Triploid Populations and Gene Ontology of a 3.3-Mb Genome Region Linked to Alternaria Brown Spot Resistance in Citrus Reveal Clusters of Resistance Genes
Source: PLoS One. 2013 Oct 8;8(10):e76755. doi: 10.1371/journal.pone.0076755 (PMC3792864; doi:10.1371/journal.pone.0076755)
Supplement: Table S6 — Annotations between 24.57 Mb (TTC8 marker) and 27.87 Mb (CiC3248-06 marker) in scaffold 3 (www.phytozome.net). (DOCX) [file pone.0076755.s006.docx]

**Table S6. Annotations between 24.57 Mb (TTC8 marker) and 27.87 Mb (CiC3248-06 marker) in scaffold 3 (www.phytozome.net)**

| Initial position (bp) | final position (bp) | Locus name | Annotations |
| --- | --- | --- | --- |
| 24584262 | 24588809 | Ciclev10023819m.g | Aluminium activated malate transporter family protein |
| 24593002 | 24597811 | Ciclev10023283m.g | **disease resistance protein (TIR-NBS-LRR class), putative** |
| 24598362 | 24600642 | Ciclev10020543m.g | alpha/beta-Hydrolases superfamily protein |
| 24611867 | 24615456 | Ciclev10024092m.g | **disease resistance protein (TIR-NBS-LRR class), putative** |
| 24617312 | 24619000 | Ciclev10021706m.g | alpha/beta-Hydrolases superfamily protein |
| 24620623 | 24627357 | Ciclev10020009m.g | Enhancer of polycomb-like transcription factor protein |
| 24629370 | 24637605 | Ciclev10018715m.g | Alkaline-phosphatase-like family protein |
| 24669795 | 24674192 | Ciclev10018914m.g | **disease resistance protein (TIR-NBS-LRR class), putative** |
| 24674735 | 24676674 | Ciclev10021413m.g | alpha/beta-Hydrolases superfamily protein |
| 24683380 | 24686055 | Ciclev10023561m.g | FAR1-related sequence 5 |
| 24687505 | 24689173 | Ciclev10023487m.g | F-box/RNI-like superfamily protein |
| 24717374 | 24720944 | Ciclev10023733m.g | **disease resistance protein (TIR-NBS-LRR class), putative** |
| 24722080 | 24724113 | Ciclev10021643m.g | alpha/beta-Hydrolases superfamily protein |
| 24726485 | 24731694 | Ciclev10021199m.g | Enhancer of polycomb-like transcription factor protein |
| 24732324 | 24734809 | Ciclev10023873m.g | Alkaline-phosphatase-like family protein |
| 24741494 | 24743967 | Ciclev10021112m.g | RHOMBOID-like protein 14 |
| 24744597 | 24750079 | Ciclev10019165m.g | DEA(D/H)-box RNA helicase family protein |
| 24750794 | 24752907 | Ciclev10022561m.g | Ribosomal L18p/L5e family protein |
| 24753302 | 24753667 | Ciclev10023727m.g | Plant self-incompatibility protein S1 family |
| 24756088 | 24758749 | Ciclev10018885m.g | cation/H+ exchanger 19 |
| 24768060 | 24768577 | Ciclev10023601m.g | NAC domain containing protein 46 |
| 24777286 | 24779149 | Ciclev10024369m.g | Ankyrin repeat family protein |
| 24779630 | 24784345 | Ciclev10018504m.g | **NB-ARC domain-containing disease resistance protein** |
| 24785318 | 24795064 | Ciclev10019018m.g | MUTL-homologue 1 |
| 24796351 | 24800588 | Ciclev10019689m.g | serine hydroxymethyltransferase 3 |
| 24802124 | 24804993 | Ciclev10022715m.g | Surfeit locus protein 5 subunit 22 of Mediator complex |
| 24806596 | 24809323 | Ciclev10023969m.g | ribosomal protein L24 |
| 24809538 | 24814289 | Ciclev10020286m.g | Transducin/WD40 repeat-like superfamily protein |
| 24815262 | 24816480 | Ciclev10023234m.g | bonsai |
| 24819124 | 24831081 | Ciclev10018511m.g | Transducin/WD40 repeat-like superfamily protein |
| 24831949 | 24837740 | Ciclev10020270m.g | methionine aminopeptidase 2B |
| 24837938 | 24840296 | Ciclev10021676m.g | Nucleic acid-binding, OB-fold-like protein |
| 24840386 | 24845254 | Ciclev10019406m.g | methylenetetrahydrofolate reductase 2 |
| 24856909 | 24862275 | Ciclev10024071m.g | histone-lysine N-methyltransferase ASHH3 |
| 24893551 | 24898675 | Ciclev10019906m.g | Peptidase family C54 protein |
| 24899223 | 24901003 | Ciclev10020147m.g | Galactose oxidase/kelch repeat superfamily protein |
| 24943610 | 24945394 | Ciclev10022372m.g | Ribosomal protein L6 family |
| 24949255 | 24954319 | Ciclev10019784m.g | **Seven transmembrane MLO family protein** |
| 24958927 | 24963659 | Ciclev10023336m.g | **Seven transmembrane MLO family protein** |
| 24966135 | 24969178 | Ciclev10024611m.g | **NB-ARC domain-containing disease resistance protein** |
| 24985138 | 24987298 | Ciclev10023902m.g | **LRR and NB-ARC domains-containing disease resistance protein** |
| 24991889 | 24992787 | Ciclev10024232m.g | **NB-ARC domain-containing disease resistance protein** |
| 24993845 | 24995370 | Ciclev10024038m.g | **NB-ARC domain-containing disease resistance protein** |
| 25026668 | 25030809 | Ciclev10024551m.g | **LRR and NB-ARC domains-containing disease resistance protein** |
| 25039430 | 25044580 | Ciclev10020313m.g | **Seven transmembrane MLO family protein** |
| 25044600 | 25051607 | Ciclev10020879m.g | protein serine/threonine kinases;ATP binding;catalytics |
| 25053425 | 25057795 | Ciclev10020158m.g | guanosine nucleotide diphosphate dissociation inhibitor 1 |
| 25067469 | 25072314 | Ciclev10019310m.g | Ankyrin repeat family protein |
| 25074163 | 25076367 | Ciclev10022861m.g | auxin-regulated gene involved in organ size |
| 25085745 | 25086451 | Ciclev10023183m.g |  |
| 25087317 | 25089114 | Ciclev10020871m.g | Late embryogenesis abundant protein, group 2 |
| 25100778 | 25104843 | Ciclev10018688m.g | **NB-ARC domain-containing disease resistance protein** |
| 25158172 | 25162574 | Ciclev10019028m.g | Prolyl oligopeptidase family protein |
| 25169542 | 25170675 | Ciclev10023618m.g |  |
| 25204996 | 25205221 | Ciclev10024113m.g | Chaperone DnaJ-domain superfamily protein |
| 25205365 | 25207063 | Ciclev10023518m.g |  |
| 25216526 | 25220629 | Ciclev10024119m.g | **LRR and NB-ARC domains-containing disease resistance protein** |
| 25248367 | 25251368 | Ciclev10023445m.g | indole-3-acetate beta-D-glucosyltransferase |
| 25269003 | 25272836 | Ciclev10018531m.g | **LRR and NB-ARC domains-containing disease resistance protein** |
| 25286483 | 25288105 | Ciclev10019912m.g | UDP-glucosyl transferase 75B2 |
| 25318178 | 25322601 | Ciclev10018594m.g | **LRR and NB-ARC domains-containing disease resistance protein** |
| 25361089 | 25365339 | Ciclev10018499m.g | **LRR and NB-ARC domains-containing disease resistance protein** |
| 25398286 | 25402615 | Ciclev10023525m.g | **NB-ARC domain-containing disease resistance protein** |
| 25426124 | 25427403 | Ciclev10024201m.g | basic helix-loop-helix (bHLH) DNA-binding superfamily protein |
| 25432861 | 25433406 | Ciclev10024286m.g | BED zinc finger ;hAT family dimerisation domain |
| 25441988 | 25444731 | Ciclev10019183m.g | RAP |
| 25445568 | 25448031 | Ciclev10023789m.g | ADP-glucose pyrophosphorylase small subunit 2 |
| 25448858 | 25451858 | Ciclev10022149m.g | 6,7-dimethyl-8-ribityllumazine synthase / DMRL synthase / lumazine synthase / riboflavin synthase |
| 25452062 | 25455844 | Ciclev10020914m.g | Dihydrodipicolinate reductase, bacterial/plant |
| 25458171 | 25463466 | Ciclev10019560m.g | Poly (ADP-ribose) glycohydrolase (PARG) |
| 25463711 | 25465970 | Ciclev10020069m.g | FBD, F-box, Skp2-like and Leucine Rich Repeat domains containing protein |
| 25470795 | 25473216 | Ciclev10020065m.g | F-box/RNI-like/FBD-like domains-containing protein |
| 25474880 | 25476924 | Ciclev10024256m.g | Thioredoxin superfamily protein |
| 25495290 | 25499648 | Ciclev10023260m.g | **LRR and NB-ARC domains-containing disease resistance protein** |
| 25539228 | 25543442 | Ciclev10018540m.g | **LRR and NB-ARC domains-containing disease resistance protein** |
| 25546089 | 25546373 | Ciclev10023953m.g | mitochondrial ribosomal protein L11 |
| 25558189 | 25563873 | Ciclev10018510m.g | **LRR and NB-ARC domains-containing disease resistance protein** |
| 25563004 | 25563956 | Ciclev10024474m.g |  |
| 25577398 | 25580479 | Ciclev10023481m.g | **NB-ARC domain-containing disease resistance protein** |
| 25591667 | 25592171 | Ciclev10022922m.g |  |
| 25596184 | 25598211 | Ciclev10019166m.g |  |
| 25598722 | 25601734 | Ciclev10020079m.g | F-box family protein |
| 25605171 | 25606024 | Ciclev10023014m.g | F-box/RNI-like superfamily protein |
| 25612741 | 25615635 | Ciclev10023374m.g | uridine-ribohydrolase 2 |
| 25625682 | 25630835 | Ciclev10019447m.g | inositol 1,3,4-trisphosphate 5/6-kinase 4 |
| 25633452 | 25636420 | Ciclev10018897m.g | **Disease resistance protein (CC-NBS-LRR class) family** |
| 25639011 | 25644826 | Ciclev10019649m.g | RNA-binding protein |
| 25645230 | 25649184 | Ciclev10021021m.g | chloroplast outer envelope protein 37 |
| 25649848 | 25653317 | Ciclev10024361m.g | S-adenosyl-L-methionine-dependent methyltransferases superfamily protein |
| 25657664 | 25663490 | Ciclev10024293m.g | Endonuclease/exonuclease/phosphatase family protein |
| 25663967 | 25668616 | Ciclev10019293m.g | Ankyrin repeat family protein |
| 25702085 | 25702716 | Ciclev10023674m.g |  |
| 25740008 | 25740471 | Ciclev10023198m.g | Pectin lyase-like superfamily protein |
| 25756520 | 25759773 | Ciclev10018637m.g | Leucine-rich repeat receptor-like protein kinase family protein |
| 25784246 | 25784449 | Ciclev10023998m.g |  |
| 25838882 | 25842061 | Ciclev10023511m.g | Leucine-rich repeat receptor-like protein kinase family protein |
| 25844828 | 25845232 | Ciclev10024127m.g | Plant self-incompatibility protein S1 family |
| 25920662 | 25925208 | Ciclev10023567m.g | Leucine-rich repeat receptor-like protein kinase family protein |
| 25929095 | 25929878 | Ciclev10024445m.g | Leucine-rich repeat receptor-like protein kinase family protein |
| 26006287 | 26009481 | Ciclev10024013m.g | Leucine-rich repeat receptor-like protein kinase family protein |
| 26027989 | 26031836 | Ciclev10024332m.g | Leucine-rich repeat receptor-like protein kinase family protein |
| 26054584 | 26055280 | Ciclev10023832m.g | Leucine-rich repeat receptor-like protein kinase family protein |
| 26061449 | 26062187 | Ciclev10023220m.g | gamma-glutamyl hydrolase 1 |
| 26288659 | 26290508 | Ciclev10021937m.g | response regulator 9 |
| 26297949 | 26304739 | Ciclev10021153m.g | Plant protein 1589 of unknown function |
| 26303573 | 26304130 | Ciclev10023069m.g |  |
| 26308767 | 26309588 | Ciclev10023742m.g | FAR1-related sequence 5 |
| 26439998 | 26440627 | Ciclev10024302m.g | FAR1-related sequence 5 |
| 26480614 | 26486649 | Ciclev10020055m.g | cystathionine beta-lyase |
| 26487045 | 26487269 | Ciclev10023935m.g | Ribosomal protein L39 family protein |
| 26517176 | 26525778 | Ciclev10019027m.g | Trimeric LpxA-like enzyme |
| 26526425 | 26531920 | Ciclev10021922m.g | tubulin folding cofactor B |
| 26552200 | 26563660 | Ciclev10018515m.g | binding |
| 26585748 | 26586352 | Ciclev10023130m.g |  |
| 26591205 | 26592308 | Ciclev10024114m.g | Glutaredoxin family protein |
| 26603126 | 26608232 | Ciclev10021999m.g | Eukaryotic rpb5 RNA polymerase subunit family protein |
| 26610747 | 26615954 | Ciclev10021431m.g |  |
| 26618336 | 26621274 | Ciclev10022600m.g | Tetratricopeptide repeat (TPR)-like superfamily protein |
| 26660974 | 26662413 | Ciclev10021720m.g | S-adenosyl-L-methionine-dependent methyltransferases superfamily protein |
| 26666978 | 26668075 | Ciclev10024496m.g | S-adenosyl-L-methionine-dependent methyltransferases superfamily protein |
| 26671216 | 26672059 | Ciclev10024436m.g | S-adenosyl-L-methionine-dependent methyltransferases superfamily protein |
| 26724892 | 26726825 | Ciclev10023900m.g | S-adenosyl-L-methionine-dependent methyltransferases superfamily protein |
| 26747398 | 26748527 | Ciclev10023903m.g | TTF-type zinc finger protein with HAT dimerisation domain |
| 26847877 | 26850055 | Ciclev10021728m.g | S-adenosyl-L-methionine-dependent methyltransferases superfamily protein |
| 26886018 | 26886467 | Ciclev10023695m.g |  |
| 26976583 | 26978658 | Ciclev10021734m.g | S-adenosyl-L-methionine-dependent methyltransferases superfamily protein |
| 27092414 | 27093612 | Ciclev10024663m.g | Zinc-binding dehydrogenase family protein |
| 27120138 | 27122618 | Ciclev10024396m.g | S-locus lectin protein kinase family protein |
| 27132311 | 27137357 | Ciclev10023678m.g | cysteine-rich RLK (RECEPTOR-like protein kinase) 8 |
| 27160750 | 27163015 | Ciclev10024040m.g | Na+/H+ antiporter 6 |
| 27174902 | 27177223 | Ciclev10023619m.g | MuDR family transposase |
| 27179651 | 27180616 | Ciclev10024633m.g |  |
| 27182222 | 27186798 | Ciclev10018865m.g | Prolyl oligopeptidase family protein |
| 27202252 | 27203036 | Ciclev10023045m.g | hemoglobin 1 |
| 27203532 | 27205968 | Ciclev10024617m.g | Zinc-binding dehydrogenase family protein |
| 27206738 | 27207496 | Ciclev10023412m.g | Zinc-binding dehydrogenase family protein |
| 27210508 | 27212925 | Ciclev10023669m.g | **NB-ARC domain-containing disease resistance protein** |
| 27218280 | 27219176 | Ciclev10023093m.g |  |
| 27222437 | 27223126 | Ciclev10022330m.g | Ribosomal protein L2 family |
| 27236430 | 27242516 | Ciclev10018492m.g | **LRR and NB-ARC domains-containing disease resistance protein** |
| 27243916 | 27244600 | Ciclev10023862m.g |  |
| 27252187 | 27258492 | Ciclev10023613m.g | **LRR and NB-ARC domains-containing disease resistance protein** |
| 27267893 | 27272240 | Ciclev10018507m.g | **LRR and NB-ARC domains-containing disease resistance protein** |
| 27274447 | 27274841 | Ciclev10023703m.g | Ribosomal protein L2 family |
| 27288919 | 27290352 | Ciclev10023361m.g | Zinc-binding dehydrogenase family protein |
| 27291189 | 27295420 | Ciclev10024454m.g | **NB-ARC domain-containing disease resistance protein** |
| 27320758 | 27322899 | Ciclev10024530m.g | **NB-ARC domain-containing disease resistance protein** |
| 27338257 | 27339658 | Ciclev10023645m.g | Ribosomal protein L2 family |
| 27365802 | 27371376 | Ciclev10018509m.g | **LRR and NB-ARC domains-containing disease resistance protein** |
| 27373577 | 27374040 | Ciclev10024425m.g | Ribosomal protein L2 family |
| 27378259 | 27383779 | Ciclev10019887m.g | Integrin-linked protein kinase family |
| 27385466 | 27391796 | Ciclev10018983m.g | LETM1-like protein |
| 27392621 | 27394211 | Ciclev10020021m.g | HXXXD-type acyl-transferase family protein |
| 27395608 | 27398058 | Ciclev10020092m.g | UDP-glucosyltransferase 74F2 |
| 27411566 | 27413326 | Ciclev10023826m.g | UDP-glucosyltransferase 74F2 |
| 27420861 | 27421928 | Ciclev10024366m.g | Protein kinase family protein with leucine-rich repeat domain |
| 27453996 | 27456865 | Ciclev10023662m.g | Leucine-rich repeat receptor-like protein kinase family protein |
| 27512875 | 27514002 | Ciclev10024240m.g | Leucine-rich repeat receptor-like protein kinase family protein |
| 27522643 | 27525763 | Ciclev10023288m.g | Leucine-rich repeat receptor-like protein kinase family protein |
| 27550207 | 27553496 | Ciclev10024387m.g | Leucine-rich repeat receptor-like protein kinase family protein |
| 27579560 | 27591874 | Ciclev10024208m.g | Leucine-rich repeat receptor-like protein kinase family protein |
| 27610925 | 27611502 | Ciclev10023474m.g | Leucine-rich repeat receptor-like protein kinase family protein |
| 27624874 | 27626398 | Ciclev10021536m.g | Protein kinase family protein with leucine-rich repeat domain |
| 27682550 | 27684073 | Ciclev10024553m.g | Protein kinase family protein with leucine-rich repeat domain |
| 27717305 | 27722483 | Ciclev10023899m.g | Protein kinase family protein with leucine-rich repeat domain |
| 27737586 | 27740735 | Ciclev10018816m.g | Leucine-rich repeat receptor-like protein kinase family protein |
| 27750059 | 27750755 | Ciclev10022950m.g | Ribosomal protein S5/Elongation factor G/III/V family protein |
| 27780847 | 27783988 | Ciclev10018798m.g | Leucine-rich repeat receptor-like protein kinase family protein |
| 27792547 | 27797748 | Ciclev10020409m.g | BTB and TAZ domain protein 3 |
| 27798973 | 27801782 | Ciclev10023071m.g | Small nuclear ribonucleoprotein family protein |
| 27802944 | 27807050 | Ciclev10019655m.g | Thioesterase/thiol ester dehydrase-isomerase superfamily protein |
| 27807172 | 27813248 | Ciclev10018774m.g | formin homology 1 |
| 27827995 | 27830405 | Ciclev10019217m.g | **Leucine-rich repeat receptor-like protein kinase family protein** |
| 27852621 | 27853757 | Ciclev10023555m.g | Eukaryotic release factor 1 (eRF1) family protein |
| 27856160 | 27856424 | Ciclev10024351m.g |  |
| 27857215 | 27861301 | Ciclev10018573m.g | **Disease resistance protein (TIR-NBS-LRR class) family** |
| 27867691 | 27874119 | Ciclev10018528m.g | **Disease resistance protein (TIR-NBS-LRR class), putative** |

Annotations related to resistance response are indicated in bold letters.
