# Supplementary material for: Genetically Based Location from Triploid Populations and Gene Ontology of a 3.3-Mb Genome Region Linked to Alternaria Brown Spot Resistance in Citrus Reveal Clusters of Resistance Genes
Source: PLoS One. 2013 Oct 8;8(10):e76755. doi: 10.1371/journal.pone.0076755 (PMC3792864; doi:10.1371/journal.pone.0076755)
Supplement: Figure S1 — Values of F parameter from ANOVA along the nine linkage groups of the ‘Clementine’ genetic map [71]. (DOCX) [file pone.0076755.s007.docx]

**Figure S1. Values of F parameter from ANOVA along the nine linkage groups of the ‘Clementine’ genetic map [71]**
